# Structural basis of a bi-functional malonyl-CoA reductase (MCR) from the photosynthetic green non-sulfur bacterium *Roseiflexus castenholzii*

**DOI:** 10.1128/mbio.03233-22

**Published:** 2023-06-06

**Authors:** Xin Zhang, Jiyu Xin, Zhiguo Wang, Wenping Wu, Yutong Liu, Zhenzhen Min, Yueyong Xin, Bing Liu, Jun He, Xingwei Zhang, Xiaoling Xu

**Affiliations:** 1 Department of Biochemistry and Molecular Biology, School of Basic Medical Sciences and the Affiliated Hospital, Key Laboratory of Aging and Cancer Biology of Zhejiang Province, Hangzhou Normal University, Hangzhou, China; 2 Photosynthesis Research Center, College of Life and Environmental Sciences, Hangzhou Normal University, Hangzhou, China; 3 CAS Key Laboratory of Regenerative Biology, Guangdong Provincial Key Laboratory of Stem Cell and Regenerative Medicine, Guangzhou Institutes of Biomedicine and Health, Chinese Academy of Sciences, Guangzhou, China; University of California, Irvine, California, USA; University of California, Irvine, California, USA

**Keywords:** malonyl-CoA reductase, short-chain dehydrogenase/reductase, *Roseiflexus castenholzii*, bi-functional enzyme, 3-hydroxypropionate

## Abstract

Malonyl-CoA reductase (MCR) is a NADPH-dependent bi-functional enzyme that performs alcohol dehydrogenase and aldehyde dehydrogenase (CoA-acylating) activities in the N- and C-terminal fragments, respectively. It catalyzes the two-step reduction of malonyl-CoA to 3-hydroxypropionate (3-HP), a key reaction in the autotrophic CO_2_ fixation cycles of *Chloroflexaceae* green non-sulfur bacteria and the archaea *Crenarchaeota*. However, the structural basis underlying substrate selection, coordination, and the subsequent catalytic reactions of full-length MCR is largely unknown. For the first time, we here determined the structure of full-length MCR from the photosynthetic green non-sulfur bacterium *Roseiflexus castenholzii* (*Rfx*MCR) at 3.35 Å resolution. Furthermore, we determined the crystal structures of the N- and C-terminal fragments bound with reaction intermediates NADP^+^ and malonate semialdehyde (MSA) at 2.0 Å and 2.3 Å, respectively, and elucidated the catalytic mechanisms using a combination of molecular dynamics simulations and enzymatic analyses. Full-length *Rfx*MCR was a homodimer of two cross-interlocked subunits, each containing four tandemly arranged short-chain dehydrogenase/reductase (SDR) domains. Only the catalytic domains SDR1 and SDR3 incorporated additional secondary structures that changed with NADP^+^–MSA binding. The substrate, malonyl-CoA, was immobilized in the substrate-binding pocket of SDR3 through coordination with Arg1164 and Arg799 of SDR4 and the extra domain, respectively. Malonyl-CoA was successively reduced through protonation by the Tyr743–Arg746 pair in SDR3 and the catalytic triad (Thr165–Tyr178–Lys182) in SDR1 after nucleophilic attack from NADPH hydrides.

**IMPORTANCE:**

The bi-functional MCR catalyzes NADPH-dependent reduction of malonyl-CoA to 3-HP, an important metabolic intermediate and platform chemical, from biomass. The individual MCR-N and MCR-C fragments, which contain the alcohol dehydrogenase and aldehyde dehydrogenase (CoA-acylating) activities, respectively, have previously been structurally investigated and reconstructed into a malonyl-CoA pathway for the biosynthetic production of 3-HP. However, no structural information for full-length MCR has been available to illustrate the catalytic mechanism of this enzyme, which greatly limits our capacity to increase the 3-HP yield of recombinant strains. Here, we report the cryo-electron microscopy structure of full-length MCR for the first time and elucidate the mechanisms underlying substrate selection, coordination, and catalysis in the bi-functional MCR. These findings provide a structural and mechanistic basis for enzyme engineering and biosynthetic applications of the 3-HP carbon fixation pathways.

## INTRODUCTION

Malonyl-CoA reductase (MCR) catalyzes NADPH-dependent reduction of malonyl-CoA to malonate semialdehyde (MSA). This is a key reaction step in two autotrophic CO_2_ fixation pathways, the 3-hydroxypropionate (3-HP) (1) and 3-HP/4-hydroxybutanoate cycles (2) that were initially identified in *Chloroflexaceae* green non-sulfur bacteria and in the archaea *Crenarchaeota*, respectively (3). Both carbon fixation pathways begin with an acetyl-CoA carboxylase (ACCase)-catalyzed carboxylation of acetyl-CoA to malonyl-CoA, which is consecutively reduced to MSA and further reduced to 3-HP. Archaeal species often utilize two mono-functional enzymes, MCR and malonate semialdehyde reductase, to catalyze malonyl-CoA conversion to 3-HP (2). In contrast, green non-sulfur bacteria, including members of the genera *Chloroflexus* and *Roseiflexus*, employ a single bi-functional MCR to catalyze both reduction reactions of malonyl-CoA (4). MSA is an electrophilic aldehyde that can form adducts to free amino groups and exert toxic effects (5). Through catalyzing two consecutive reduction reactions, the bi-functional MCR directly converts MSA into 3-HP, thereby decreasing the cytotoxicity that resulted from the accumulation of this aldehyde intermediate. Given the important role of 3-HP as one of the 12 value-added platform chemicals generated from biomass (6, 7), a malonyl-CoA pathway for 3-HP biosynthesis containing ACCase and the bi-functional MCR has been reconstructed in *Escherichia coli*, yeast, *Synechocystis*, and type II methanotrophs (8 - 12). However, all recombinant strains carrying the malonyl-CoA pathway have low 3-HP yield, which was resulted from the imbalance in expression levels, catalytic activities, and metabolic coordination of the multi-subunit ACCase and bi-functional MCR enzymes (13, 14).

*Chloroflexus aurantiacus* is a representative species of green non-sulfur bacteria in which the 3-HP cycle was originally identified (15, 16). The MCR enzymes purified from both autotrophically and heterotrophically grown *C. aurantiacus* cells (*Cfx*MCR) have been dissected into two functionally distinct fragments, *Cfx*MCR-N and *Cfx*MCR-C, which perform the alcohol dehydrogenase and aldehyde dehydrogenase (CoA-acylating) activities, respectively (4). Malonyl-CoA is primarily reduced to MSA by *Cfx*MCR-C, then further reduced to 3-HP by *Cfx*MCR-N. Dissection of *Cfx*MCR into these two individual fragments not only increases the overall enzyme activity but also improves 3-HP production in recombinant *E. coli* strains (9, 12, 17). However, *Cfx*MCR-N is expressed at significantly higher levels than *Cfx*MCR-C in 3-HP-producing strains, even though both genes are codon-optimized and controlled by the same promoter (17). In combination with the rate-limiting effects of reduction catalyzed by MCR-C, the difference in expression generates a functional imbalance between MCR-N and MCR-C, which further decreases the 3-HP yield (17). Direct evolution of *Cfx*MCR-C and fine-tuning of *Cfx*MCR-N expression levels can minimize the imbalance of overall enzyme activity, which increased the 3-HP titer 270-fold in one instance (18); however, even when this approach is combined with optimized culture conditions, 3-HP production remains unsatisfactory. Structural and mechanistic investigations into the bi-functional MCR are, therefore, urgently needed.

Crystal structures of individual MCR-N and MCR-C from *Porphyrobacter dokdonensis* have revealed dimeric architectures of both *Pd*MCR-N and *Pd*MCR-C, which fit well into the surface model of the full-length *Pd*MCR resolved from small-angle X-ray scattering (SAXS) analyses. It is proposed by the authors that a full-length *Pd*MCR dimer was formed through paring contact of two subunits, with each subunit composed of four tandemly arranged short-chain dehydrogenase/reductase (SDR) domains (19). However, due to the lack of a high-resolution structure of full-length MCR, the assembly of the homodimer, as well as the conformation of MCR-N and MCR-C connecting regions are not clear. Although *Pd*MCR-N and *Pd*MCR-C structures revealed the cofactor/substrate-binding mode, the molecular mechanisms underlying substrate selection, intermediate coordination, and subsequent catalytic processes of bi-functional MCR are not fully understood. These greatly limit our understanding of the catalytic mechanisms of bi-functional MCRs and also the potential to engineer a full-length enzyme for improving biosynthetic 3-HP production.

For the first time, we here determined the cryo-electron microscopy (EM) structure of the full-length MCR from *Roseiflexus castenholzii* (20) (*Rfx*MCR), which shares 58% sequence identity with *Cfx*MCR (21), at 3.35 Å resolution. The crystal structures of NADP^+^–MSA-bound *Rfx*MCR-N and *Rfx*MCR-C were also determined at 2.0 Å and 2.3 Å resolutions, respectively. Full-length *Rfx*MCR was a homodimer of two cross-interlocked subunits that each contained four tandemly arranged SDR domains; only the catalytic SDR1 and SDR3 domains, which incorporated additional components (such as the α10 helix and the ED) into the SDR core, were capable of accommodating NADP^+^–MSA as reaction intermediates. Molecular dynamics (MD) simulations of the full-length *Rfx*MCR further revealed that malonyl-CoA binding required the cooperation of Arg1164 from SDR4 and Arg799 from the ED. Malonyl-CoA was successively reduced through a proton-relay network formed by the Tyr743-Arg746 pair in SDR3 and the catalytic triad Thr165-Tyr178-Lys182 in SDR1 after nucleophilic attack by the NADPH hydrides. Our work illustrates the molecular mechanisms underlying substrate selection, binding, and consecutive reduction by the bi-functional enzyme MCR. These findings will serve as the structural basis for future enzyme engineering and biosynthetic applications of the malonyl-CoA pathway for 3-HP production.

## MATERIALS AND METHODS

### Protein expression and purification

The gene sequences encoding *R. castenholzii* MCR (*Rcas*_2929) was amplified from *R. castenholzii* DSM 13941 genomic DNA and inserted into pET20b expression vector at *XhoI* and *NdeI* to construct the C-terminal His_6_-tagged expression vector. The sequenced plasmid was transformed into *E. coli* BL21(DE3) cells for the recombinant expression of a C-terminal His_6_-tagged full-length *Rfx*MCR. The gene sequences encoding *Rfx*MCR-N (Met1-Phe572) and *Rfx*MCR-C (Gly573-Val1229) were inserted into pET28a vectors at *XhoI* and *NcoI* restriction site, and *NdeI* and *NcoI* restriction site, respectively, to express the C-terminal and N-terminal His_6_-tagged recombinant proteins. The transformed cells were grown in 1 L Luria-Bertani broth containing 100 mg/mL ampicillin at 37°C until the optical density at 600 nm (OD_600_) reached 0.6–0.8. The gene expression was then induced with 0.2 mM isopropyl-β-D-thiogalactopyranoside overnight at 25°C.

Cells were harvested by centrifugation at 7,500× *g* for 10 minutes at 4°C and resuspended in wash buffer containing 50 mM Tris-HCl pH 8.0, 300 mM NaCl, and 2 mM MgCl_2_ prior to homogenization with a high-pressure homogenizer (Union, People’s Republic of China). The insoluble cell debris was removed by centrifugation at 22,000× *g* for 40 minutes at 4°C. The supernatant containing crude soluble proteins was loaded onto a Ni^2+^-chelating affinity chromatography column (GE Healthcare, Fairfield, CT, USA) and was rinsed with 100 mL of binding buffer (50 mM Tris-HCl pH 8.0, 300 mM NaCl, 2 mM MgCl_2_, 50 mM imidazole) to remove non-specifically bound proteins. The bound full-length *Rfx*MCR, *Rfx*MCR-N, and *Rfx*MCR-C proteins were eluted with the binding buffer containing 300 mM imidazole. The eluates were further purified by a HiLoad 16/600 Superdex 200PG size exclusion column (GE Healthcare) with buffer containing 25 mM Tris-HCl pH 8.0 and 150 mM NaCl to 95% purity.

### Cryo-electron microscopy

Three-microliter aliquots of full-length *Rfx*MCR (0.3 mg/mL) was placed on the glow-discharged GiG R1.2/1.3 300-mesh gold holey nickel titanium grid (Zhenjiang Lehua Electronic Technology, China) and blotted for 3.0 seconds under a blot force of 4 at 100% humidity and 16°C before being flash-frozen in liquid ethane with a Mark IV Vitrobot system (FEI). Micrographs were acquired on a Titan Krios microscope (FEI) operated at 300 kV with a K3 Summit direct electron detector (Gatan). SerialEM (22) was used for automatic data collection. A nominal magnification of ×22,500 was used for imaging, which yielded a pixel size of 1.07 Å. The defocus range was between 1.3 and 1.8 µm. Each micrograph was dose-fractionated to 32 frames under a dose rate of 9.4 e^-^/Å^2^ per second and an exposure time of 6.4 seconds, which resulted in a total dose of about 60 e^-^/Å^2^.

### Image processing

Motion correction and exposure weighting were performed by the MotionCorr2 program (23), and the CTF (contrast transfer function) parameter was estimated using the CtfFind program (24). All the image processing steps were performed using RELION 3.0 (25) and CryoSPARC programs (26). To generate a template for two-dimensional (2D) classification, 14,112 particles were auto-picked from 20 micrographs and subjected to 2D classification. Using a reference generated from 2D classification of 12,105 particles, 4,043,667 particles were auto-picked from 4,647 micrographs and imported in CryoSPARC. After five iterative rounds of 2D classifications, 2,391,743 particles were selected and imported in RELION for three-dimensional (3D) classification. Using the best 3D class as a reference, 3D classifications generated four classes of particles. Then 1,766,605 particles from the best two classes (percentage of 42.89 and 30.97, respectively) were selected and extracted, and subjected to another round of reference-based 3D classiﬁcation and non-uniform reﬁnement in CryoSPARC, which produced an EM-map with a global resolution of a 3.35 Å based on the gold standard Fourier shell correlation (FSC). Local resolution was estimated with Resmap (27) (Fig. S2).

### Model building, refinement, and validation

Based on the cryo-EM density map, *de novo* atomic model building of *Rfx*MCR was conducted in Coot (28). Then real-space refinement in PHENIX (29, 30) was used for model refinement. All figures were drawn in PyMOL (The PyMOL Molecular Graphics System, Version 2.5.2; Schrödinger, LLC), USCF chimera (31), or ChimeraX (32). The refinement statistics were summarized in Table 2.

### Crystallization of NADP^+^–MSA-bound *Rfx*MCR-N and *Rfx*MCR-C

The purified R*fx*MCR-N and *Rfx*MCR-C were concentrated using an Amicon Ultra centrifugal filter device (10-kDa molecular weight cutoff; Millipore) at 4°C. Protein concentrations were determined using a NanoDrop device (IMPLEN) by recording the absorption at 280 nm. The protein samples were diluted to 25 mg/mL in buffer (50 mM Tris-HCl pH 8.0, 150 mM NaCl) for crystallization. The *Rfx*MCR-N and *Rfx*MCR-C were incubated with cofactor NADP^+^ at 1: 10 molar ratio for 30 seconds at 50°C before crystallization. Crystallization was performed using the hanging-drop vapor diffusion method, with 1.2 µL of protein sample mixed with an equal volume of reservoir solution, and the mixture was equilibrated against 200 µL reservoir solution. Crystals of NADP^+^–MSA-bound *Rfx*MCR-N were obtained with the reservoir solution containing 1 M sodium malonate pH 5.0, 0.1 M sodium acetate trihydrate pH 4.5, and 3% polyethylene glycol 20,000 at 16°C. The NADP^+^–MSA-bound *Rfx*MCR-C were crystallized with reservoir solution containing 1.6 M sodium formate and 0.1 M Bis-tris propane pH 7.0 at 16°C.

### Crystal data collection, structure determination, and refinement

The optimized crystals were cryo-protected by adding 30% glycerol to the reservoir solution and flash-freezing with liquid nitrogen. A 2.0-Å data set of NADP^+^–MSA-bound *Rfx*MCR-N and a 2.30-Å data set of NADP^+^–MSA-bound *Rfx*MCR-C were collected at SSRF BL10U2 (Table 3). Diffraction data were automatically processed, integrated, and scaled with Porpoise XDS software (33). The quality of the data were assessed using SFCHECK (34), and the solvent content was calculated using Matthews_Coef from the CCP4 package (35, 36). The NADP^+^–MSA-bound *Rfx*MCR-N and *Rfx*MCR-C structures were determined by molecular replacement method using the cryo-EM structural model of full-length *Rfx*MCR as a search model. The Phaser program (37) from the CCP4 package was employed to determine the initial phases; iterative model building and refinement were performed using Coot (28), Refmac5 (38), and Phenix (29) to obtain the refined model (Table 3).

### Site-directed mutagenesis and enzyme activity assay

Site-directed mutagenesis of the catalytic triad and amino acid residues that are involved in NADP^+^–MSA binding was performed using a mutagenesis kit (Yeasen), the constructed plasmids containing the mutated gene sequences were sequenced, and transformed into *E. coli* BL21(DE3) cells for the expression of the mutant proteins. The proteins were expressed and purified following the same procedure under identical conditions as the wild-type proteins.

The enzymatic activity of *Rfx*MCR was assayed spectrophotometrically at 50°C by measuring the absorbance of NADPH at 340 nm (*ε* = 6.22 mM^-1^·cm^-1^). The standard assay mixture (200 µL) was composed of 100 mM Tris-HCl pH 8.0, 150 mM NaCl, 5 mM MgCl_2_, 0.4 mM NADPH, and 0.1 nmol *Rfx*MCR as the enzyme. Enzyme concentration was determined by the Bradford method. The absorbance of NADPH at 340 nm was recorded for 10 minutes at 50°C. One unit of enzymatic activity was deﬁned as the amount of enzyme that catalyzes the oxidation of 1 µmol NADPH per minute. The apparent Michaelis–Menten constant (*K*_m_) and *V*_m_ were measured at the reaction velocity by varying the substrate malonyl-CoA concentrations at 0.05, 0.1, 0.15, 0.2, 0.4, 0.5, and 1.0 mM (Table 1). The *k*_cat_ and *K*_m_ values were determined by the non-linear least squares fitting method. The enzymatic activity of the site-directed mutants was measured in the same conditions as the wild-type *Rfx*MCR. All the enzymatic data were obtained from triplicate experiments.

**TABLE 1:** Kinetic parameters of the full-length *Rfx*MCR^*a*^

	*K*_m_ (mM)	*k*_cat_ (s^−1^)	*k*_cat_/*K*_m_ (mM^−1^ s^−1^)
***Rfx*MCR**	0.38 ± 0.08	5.65 ± 0.57	14.8 ± 1.54

^*a*^ The kinetic parameters were determined under the optimal conditions. Data represent mean ± SD (*N* = 3).

### Sedimentation velocity analytical ultracentrifugation

Sedimentation velocity analytical ultracentrifugation (AUC) was performed to check the oligomerization state of *Rfx*MCR-N and *Rfx*MCR-C in solution. Sedimentation experiments were performed on a Beckman Coulter Proteome Lab XL-I ultracentrifuge using a 4-hole An-60Ti rotor. Protein samples with an initial absorbance at 280 nm of approximately 0.5–0.8 were equilibrated for 2 hours at 20°C under a vacuum prior to sedimentation. The absorbance at 280 nm was measured using a continuous scan mode during sedimentation at 55,000 rpm in 12 mm double-sector cells. The data were analyzed using sedfit (39).

### Molecular dynamics simulation and binding free-energy calculations

To obtain the binding structure of full-length *Rfx*MCR with the substrate malonyl-CoA, the structures of full-length *Rfx*MCR and NADP^+^–MSA-bound *Rfx*MCR-C were superimposed with the CoA bound *Pd*MCR-C (PDB 6K8T) (19) to construct the binding structure of NADP^+^–CoA–MSA-bound full-length *Rfx*MCR. Then, the structure of full-length *Rfx*MCR in complex with NADPH–malonyl-CoA was obtained through structure editing using the UCSF ChimeraX software (32). The missing residues in the determined structures were added with UCSF ChimeraX as well. To investigate the binding features of full-length *Rfx*MCR in complex with the reaction intermediate NADP^+^–MSA, and the substrate for the second reduction reaction NADPH–MSA, the binding structures of full-length *Rfx*MCR with NADP^+^–MSA and NADPH–MSA were constructed based on the crystal structures of NADP^+^–MSA-bound *Rfx*MCR-C and *Rfx*MCR-N, respectively.

To characterize the binding features of full-length *Rfx*MCR with malonyl-CoA and MSA, MD simulations were performed on the binding structures of full-length *Rfx*MCR bound with NADPH–malonyl-CoA, NADP^+^–MSA (NADP^+^–MSA bound at the C-terminal domain of full-length *Rfx*MCR), and NADPH–MSA (NADPH–MSA bound at the N-terminal domain of full-length *Rfx*MCR) by using the AMBER 20 software (40). Each binding complex was immersed into the center of a truncated octahedron box of TIP3P water molecules with a margin distance of 12.0 Å. Environmental sodium counterions were added to keep the system in electric neutrality. The AMBER ff14SB force field was applied for full-length *Rfx*MCR (41). The force field parameters of NADPH and NADP^+^ were retrieved from previous reports (42, 43). For malonyl-CoA, the atomic partial charges were calculated using the restricted electrostatic potential method with a basis set of HF/6-31G(d) on the structures optimized at the B3LYP/6-31G(d) level (44). The other force field parameters of malonyl-CoA were generated from the Generalized Amber Force Field with the Antechamber module of AmberTools (45). Following the same procedure in our previous report (46), each MD simulation was conducted with a time scale of 100 ns.

To evaluate the binding affinities between full-length *Rfx*MCR and the bound substrate and cofactors, their binding free energies (Δ*G*_bind_) were obtained through the molecular mechanics/generalized Born surface area (MM/GBSA) calculation approach (47):


(1) $$\Delta G_{bind}=G_{complex}-(G_{protein}+G_{ligand})$$

(2) $$\Delta G_{bind}=\Delta H-T\Delta S\approx \Delta E_{MM}+\Delta G_{solv}-T\Delta S$$

(3) $$\Delta E_{MM}=\Delta E_{vdW}+\Delta E_{ele}$$

(4) $$\Delta G_{solv}=\Delta G_{GB}+\Delta G{}_{SA}$$

where *E*_MM_ is the gas phase interaction energy comprising van der Waals energy (*E*_vdW_) and electrostatic energy (*E*_ele_). *G*_solv_ is the solvation free energy, including the contributions form a polar part (*G*_GB_) and a non-polar part (*G*_SA_). Δ*G*_GB_ was estimated using the generalized Born model with the interior and exterior dielectric constants set to 4 and 80, respectively (48). Δ*G*_SA_ was estimated using the LCPO algorithm: Δ*G*_SA_ = γΔ*SASA* + β, where γ and β were set to 0.0072 and 0, respectively (49). The solute entropy term TΔ*S* is sometimes approximated by normal mode entropy (50), but such treatment rarely leads to improvement in the correlation with experiments (51). Therefore, the solute entropy term was not included in the current study. In calculating Δ*G*_bind_, 200 snapshots were evenly extracted from the last 20 ns trajectories for the calculations of Δ*E*_vdW_, Δ*E*_ele_, Δ*G*_GB_, and Δ*G*_SA_.

## RESULTS

### Cryo-EM structure of full-length *Rfx*MCR

**Fig 1:**
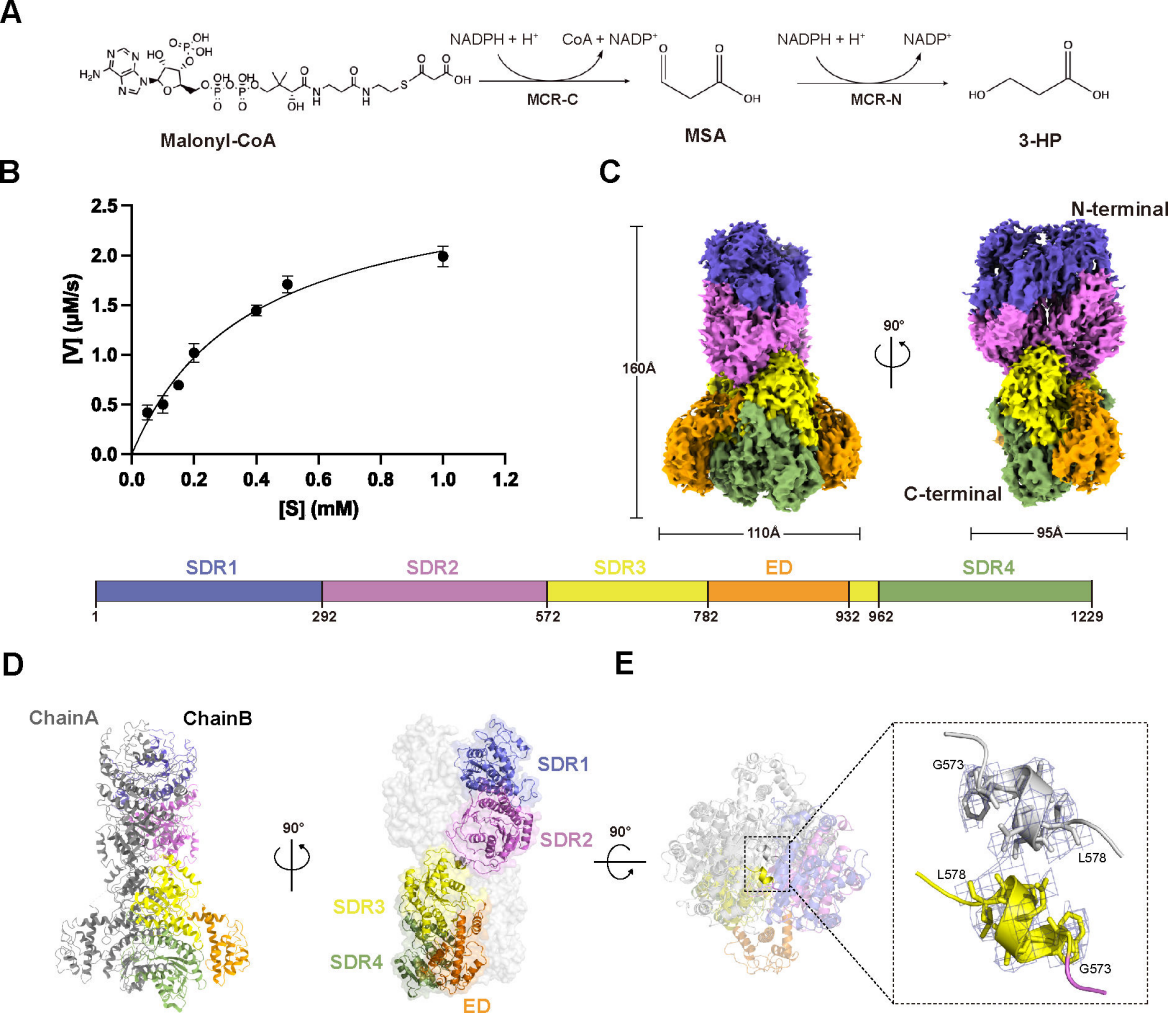
Full-length malonyl-CoA reductase (MCR) structure as determined from cryo-electron microscopy (EM) of *Roseiflexus castenholzii* MCR (*Rfx*MCR). (**A**) MCR catalyzes a two-step reduction of malonyl-CoA to malonate semialdehyde (MSA) and further reduction of MSA to 3-hydroxypropanoate (3-HP). (**B**) Full-length *Rfx*MCR kinetics. Reaction velocity was plotted from a range of starting malonyl-CoA concentrations to calculate the apparent kinetic constants of malonyl-CoA. (**C**) A cryo-EM map of full-length *Rfx*MCR shown from the front (left) and the back (right) views, represented with overall dimensions of the homodimer. Each monomer contains four tandemly arranged short-chain dehydrogenase/reductase (SDR) domains (one each in blue, violet, yellow, and sage) and one extra domain (ED) (orange) that is inserted into SDR3. The primary structure is depicted below the models to indicate domain organization. (**D**) Cartoon representation of the full-length *Rfx*MCR homodimer, which is composed of two cross-interlocked subunits. One subunit of the dimer is colored in gray, and the other uses the same color scheme as in panel C. (**E**) The top view of the full-length *Rfx*MCR homodimer (left), and the two anti-parallel arranged α-helices (G^573^WAESL^578^) that are located in the point of intersection (right). Stick models of the amino acid residues contained in these two helices are shown and labeled (right).

The gene sequence (*Rcas*_2929) encoding the full-length *Rfx*MCR (Met1-Val1229) was expressed with a C-terminal His_6_-tag in *E. coli* BL21 (DE3) cells (17). The encoded recombinant protein was purified via nickel-nitriloacetic acid affinity and size exclusion chromatography (Fig. S1A and B). Gel filtration analysis of purified *Rfx*MCR revealed an elution peak at 59.8 mL (Fig. S1A), which corresponded to the elution profile of an *Rfx*MCR dimer with a calculated molecular weight of ~270 kD. Additionally, a single protein band with a molecular weight near 268 kDa was observed in the gel after Native PAGE (Fig. S1C), confirming the existence of the *Rfx*MCR dimer in solution. Using malonyl-CoA as the substrate and NADPH as the cofactor, purified full-length *Rfx*MCR was able to catalyze the reduction of malonyl-CoA to 3-HP (Fig. 1A and B). The apparent catalytic constant (*k*_cat_) was 5.65 ± 0.57 per second and the Michaelis constant (*K*_m_) was 0.38 ± 0.08 mM for malonyl-CoA (Table 1). Compared to *Cfx*MCR, *Rfx*MCR exhibited a lower catalytic efficiency for malonyl-CoA reduction, characterized by the decreased substrate-binding affinity and turnover number (17). This difference may be attributed to the varied amino acid residues that affect the conformation of substrate-binding pocket or resulted from the different temperature and pH values for measuring the enzyme activity.

Prior to our study, the structure of full-length MCR had not been reported. To investigate the dimer structure, *Rfx*MCR was imaged on a 300 kV Titan Krios cryo-EM with a K3 Summit direct electron detector (Gatan) in counting mode (Fig. S1D). From 4,647 micrographs, 4,043,667 particles were selected and used to construct an electron potential map with an average resolution of 3.35 Å (Fig. S1D through G; Fig. S2). The final reconstructed cryo-EM map was clearly resolved and enabled us to build an accurate model of the protein side chains of full-length *Rfx*MCR (Table 2; Fig. S3A). The overall structure of *Rfx*MCR was a homodimer of two cross-interlocked subunits with the N- and C-terminal ends assembled in the same direction; the dimensions were 160 × 110 × 95 Å (Fig. 1C). Each subunit was composed of four tandemly arranged SDR domains (SDR1–4). The ED (Gly783–Arg932), which comprised eight α-helices, was inserted into the SDR3 domains (Gly573–Glu782 and Met933–Gly962) and was exposed at the two sides of the homodimer (Fig. 1C and D). Each SDR domain adopted a typical Rossmann fold that is composed of a central seven-stranded parallel β-sheet sandwiched with two or three α-helices (Fig. S3B).

**TABLE 2:** Cryo-EM data collection and refinement statistics of full-length *Rfx*MCR

Data collection and processing	
Voltage (kV)	300
Detector	K3 Summit (Gatan)
Magnification	22,500
Pixel size (Å)	1.07
Defocus range (μm)	1.3–1.8
Electron exposure (e^−^/Å^2^)	60
Symmetry imposed	*C2*
Initial particle images (*n*)	4,043,667
Final particle images (*n*)	1,766,605

Refinement	
Map resolution (Å)	3.35
FSC threshold	0.143
Model composition	
Chains	2
Non-hydrogen atoms	17,499
Protein residues	2,368
Ligands	0
Root mean square deviations	
Bond lengths (Å) Bond angles (°)	0.007 1.120
Ramachandran plot	
Favored (%)	91.91
Allowed (%)	8.09
Outliers	0.00
Validation	
MolProbity score	2.01
Clashscore	9.91
Rotamer outliers (%)	0.35

From the full-length *Rfx*MCR structure, we first resolved the conformation of *Rfx*MCR-N and *Rfx*MCR-C connecting region, which was folded into an α-helix (G^573^WAESL^578^). In the center of the homodimer, two of these α-helices were anti-parallel arranged in the reverse direction (Fig. 1E). Gel filtration and Native-PAGE analyses of the site-directed mutants W574A/E576A/F579A showed the same dimer formation as wild-type *Rfx*MCR (Fig. S1H and I), indicating that this helix is not essential for forming the homodimer. Instead, it plays an important role in connecting the *Rfx*MCR-N and *Rfx*MCR-C fragments and also serves as a point of intersection for the two subunits to form a dimer. This also confirmed that the two subunits were cross-interlocked to form the homodimer; this finding was inconsistent with the simulated SAXS model of *Pd*MCR, in which the homodimer is formed by two parallel-contacted subunits (19). Actually, the dimer interface was formed by symmetrically distributed hydrogen bonding interactions between amino acid residues from the *Rfx*MCR-N (SDR1–SDR2) and *Rfx*MCR-C (SDR3–ED–SDR4) of the two subunits (Fig. S4A and B).

### Crystal structure of substrate-bound *Rfx*MCR-N and *Rfx*MCR-C

**Fig 2:**
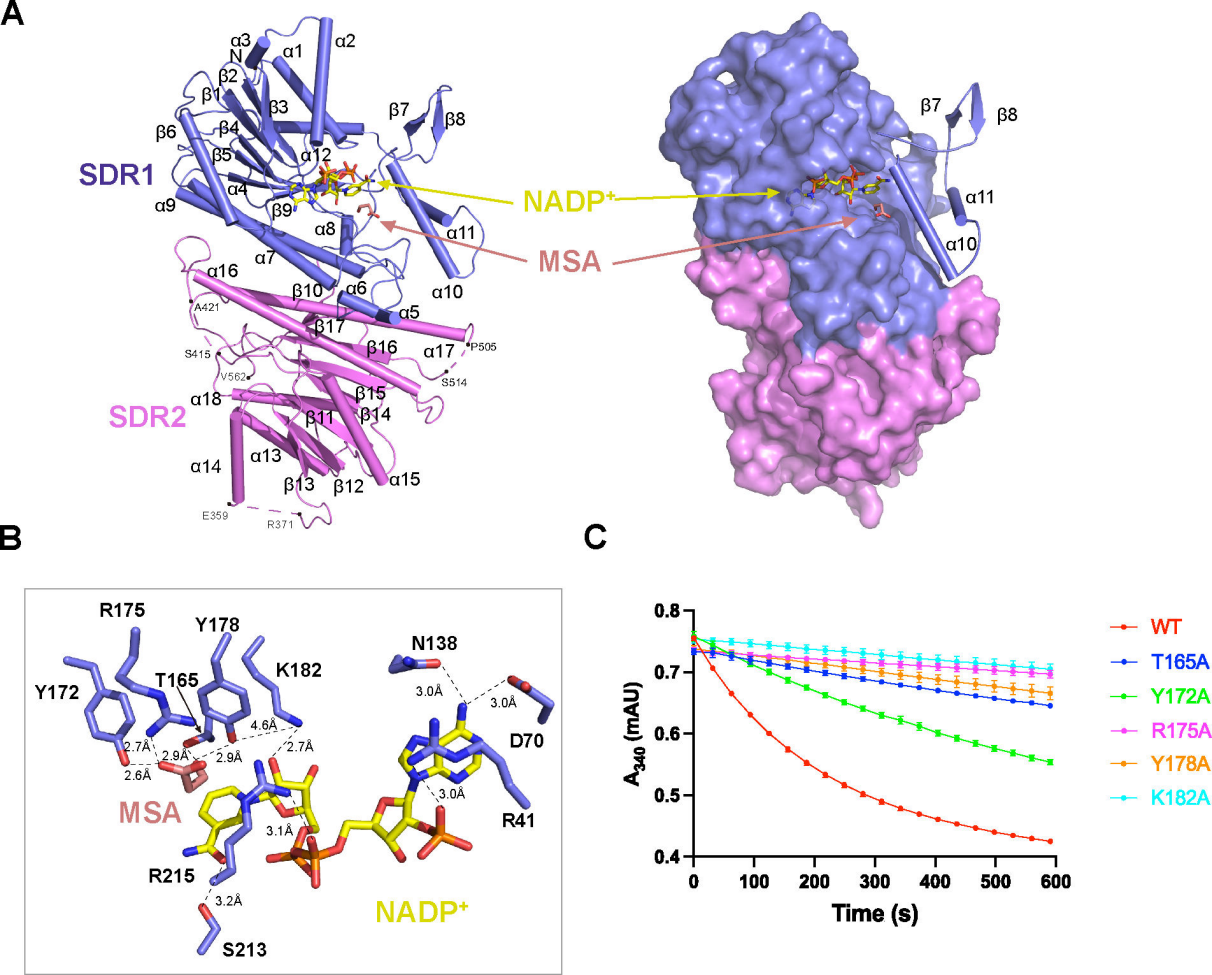
Crystal structure of the *Roseiflexus castenholzii* malonyl-CoA reductase (*Rfx*MCR) N-terminal region bound with NADP^+^ and malonate semialdehyde (MSA). (**A**) Overall structure of NADP^+^–MSA-bound *Rfx*MCR-N is shown as ribbon (left) and surface models (right). The cofactor NADP^+^ (shown in yellow) and the reaction intermediate MSA (shown in salmon) are resolved in the substrate-binding pocket of the SDR1 domain (blue), but not in the SRD2 domain (violet). The regions in SDR2 without a clear electron density map (Glu359-Arg371, Ser415-Ala421, and Pro505-Ser514) are labeled and indicated with dashed lines. The α10 and α11 helices, which cover the NADP^+^–MSA binding site, are shown as ribbons. (**B**) NADP^+^–MSA binding in the *Rfx*MCR-N substrate-binding pocket. The amino acid residues coordinating NADP^+^ (yellow) and MSA (salmon) are labeled and shown in stick form. Hydrogen-bonding interactions are labeled with the corresponding distances and indicated with dashed lines. (**C**) Overall enzymatic activity of mutant forms of full-length *Rfx*MCR. Members of the catalytic triad (Thr165–Tyr178–Lys182) and Tyr172 and Arg175, which are necessary for coordinating MSA, were mutated to Ala residues. All the enzymatic data were obtained from triplicate experiments.

**Fig 3:**
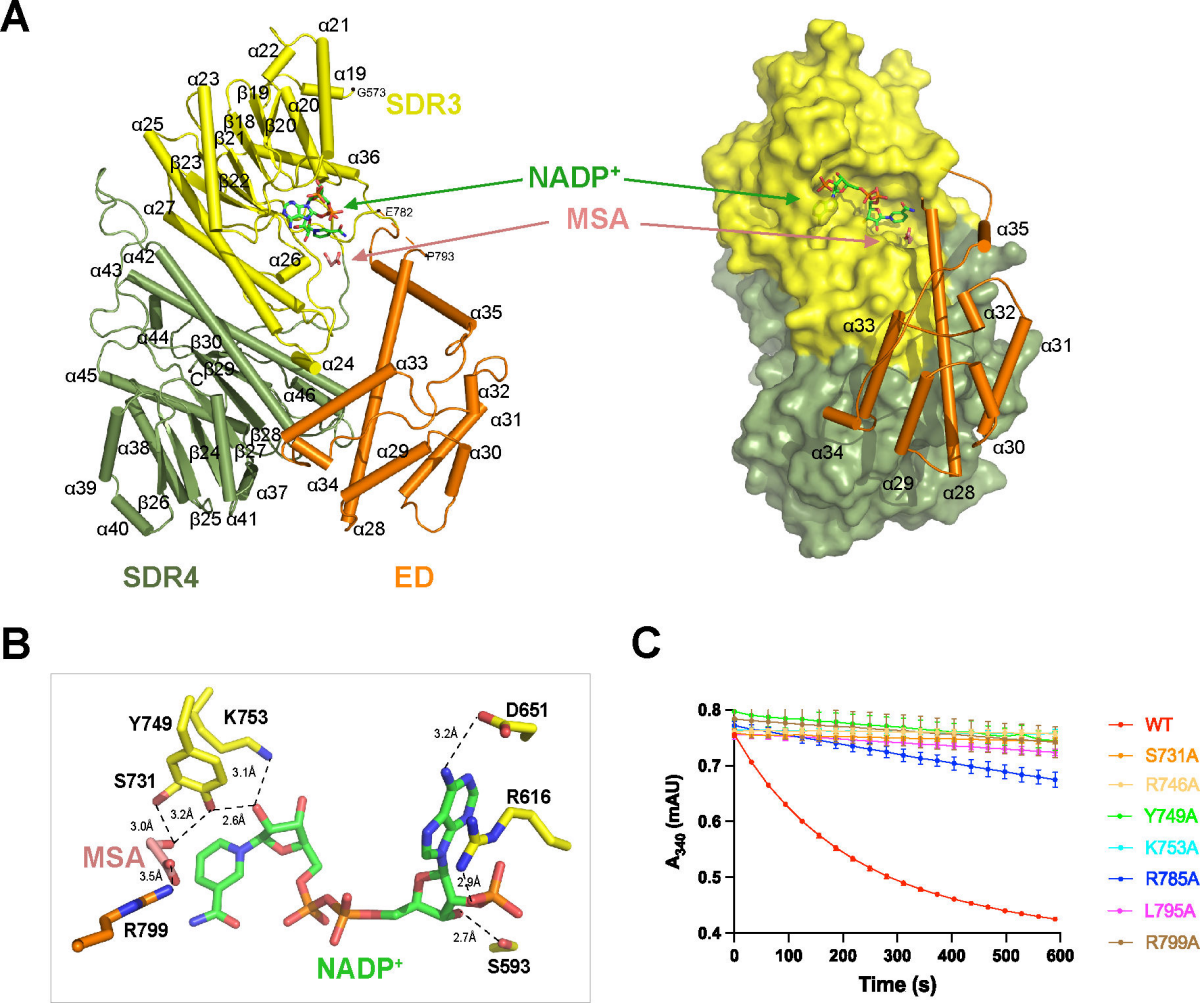
Crystal structure of the *Roseiflexus castenholzii* malonyl-CoA reductase (*Rfx*MCR) C-terminal region bound with NADP^+^ and malonate semialdehyde (MSA). (**A**) Overall structure of NADP^+^–MSA-bound *Rfx*MCR-C is shown as ribbon (left) and surface models (right). The extra domain (ED) is shown in ribbon form to demonstrate its role in forming the substrate-binding pocket. The cofactor NADP^+^ (shown in green) and the reaction intermediate MSA (shown in salmon) are coordinated in the substrate-binding pocket formed by the SDR3 domain (yellow), the ED (orange), and the SRD4 domain (sage). The region in the ED without a clear electron density map (Glu782–Pro793) is labeled and indicated with a dashed line. (**B**) Coordination of NADP^+^–MSA in the substrate-binding pocket of *Rfx*MCR-C. The amino acid residues coordinating NADP^+^ (green) and MSA (salmon) are shown as stick models. Dashed lines indicate hydrogen bonding interactions. (**C**) Enzymatic analyses of mutant forms of full-length *Rfx*MCR. Several residues were mutated to Ala residues: members of the catalytic triad (Ser731–Lys753–Tyr749); Arg799, which is necessary for coordinating MSA; and the highly conserved amino acid residues Arg746, Arg785, and Leu795, which are essential for CoA binding in *Porphyrobacter dokdonensis* MCR. All the enzymatic data were obtained from triplicate experiments. WT, wild type.

Attempts to obtain a cryo-EM structure of substrate-bound full-length *Rfx*MCR were not successful. We did not resolve any densities of substrate or cofactor from the cryo-EM maps of full-length *Rfx*MCR incubated with NADP^+^ or malonyl-CoA (Fig. S3E and F). To elucidate the substrate binding and catalytic mechanisms of *Rfx*MCR, we therefore determined the crystal structures of *Rfx*MCR-N (Met1–Phe572) and *Rfx*MCR-C (Gly573–Val1229) bound with NADP^+^–MSA (Fig. 2 and 3). The crystal structure of *Rfx*MCR-N in complex with NADP^+^–MSA was determined by the molecular replacement method and reﬁned to an *R*_work_ of 19.62% and an *R*_free_ of 20.81% at 2.0 Å resolution (Table 3). Although the *P6_5_22* crystal contained only a monomer *Rfx*MCR-N in one asymmetric unit, it was packed into a homodimer in the crystal (Fig. S4C). This was consistent with both the gel filtration and AUC analyses, which showed the existence of an *Rfx*MCR-N dimer in solution (Fig. S5A and D). Superimposition of the crystal-packed *Rfx*MCR-N dimer with the full-length *Rfx*MCR yielded a root mean square deviation (RMSD) of 1.031 Å (Fig. S4D), indicating that *Rfx*MCR-C truncation did not affect the overall conformation of *Rfx*MCR-N. Analyses of the dimer interface revealed similar symmetrically distributed hydrogen bonding interactions as the full-length *Rfx*MCR dimer, such as those between Arg6 and Glu274 (2.9 Å), Glu190 and Arg243 (3.0 Å), Ala277 and His287 (2.9 Å), and Thr297, Thr298, and Glu552 (2.9 Å) (Fig. S4A).

**TABLE 3:** X-ray diffraction data collection and structure refinement statistics of the NADP^+^–MSA-bound *Rfx*MCR-N and *Rfx*MCR-C

	NADP^+^–MSA-bound *Rfx*MCR-N	NADP^+^–MSA-bound *Rfx*MCR-C
**Data collection**		
Diffraction source	BL10U2, SSRF	BL10U2, SSRF
Wavelength (Å)	0.9792	0.9792
Space group	*P6_5_22*	*P6_5_22*
Cell parameters (Å)	*a* = 98.19, *b* = 98.19, *c* = 333.54	*a* = 83.67, *b* = 83.67, *c* = 375.54
	*α* = *β* = 90.0°, *γ* = 120.0°	*α* = *β* = 90.0°, *γ* = 120.0°
Resolution (Å)	42.31 (2.07)^*a*^ − 2.00	41.58 (2.38) − 2.30
Total reflections	2,482,704 (85,143)	1,342,488 (136,755)
Unique reflections	65,288 (2,754)	35,934 (3,517)
*R*_merge_ (%)^*b*^	0.2887 (2.654)	0.1298 (1.563)
*I*/*σ* (*I*)	19.95 (3.05)	22.39 (3.85)
Completeness (%)	99.92 (99.85)	99.92 (100.00)
**Refinement**		
Resolution (Å)	42.31 (2.07)^*a*^ − 2.00	41.58 (2.38) − 2.30
*R*_work_/*R*_free_ (%)	19.62/20.81	22.05/25.28
Root mean square deviations		
Bonds (Å)	0.007	0.007
Angles (°)	0.86	0.91
Wilson B-factor	29.88	48.90
Average B-factor	32.24	55.47
Ramachandran plot		
Favored (%)	97.34	97.98
Allowed (%)	2.47	2.02
Outliers (%)	0.19	0.00

^*a*^ Values in parentheses are for the highest-resolution shell.

^*b*^ *R*_merge_ = ∑_*hkl*_ ∑_i_ │*I*_*i*_(*hkl*) -〈*I*(*hkl*)〉│/ ∑_*hkl*_ ∑_*i*_ *I*_*i*_(hkl), where *I*_*i*_(*hkl*) is the intensity of the *i*th measurement of reflection *hkl* and 〈*I*(*hkl*)〉 is the mean intensity of all symmetry-related reflections.

Although SDR1 (Met1–Pro292) and SDR2 (Thr293–Gly572) in *Rfx*MCR-N had identical architectures, only SDR1, which contained the conserved Tyr-X-X-X-Lys motif (52), was resolved with NADP^+^ bound (Fig. 2A; Fig. S6A). Surprisingly, we observed an extra electron density that precisely matched the reduction intermediate MSA near the nicotinamide ring of NADP^+^ (Fig. S3C). The carboxyl groups of MSA were immobilized by hydrogen bonding interactions with Tyr172 (2.6 Å), Arg175 (2.7 Å), and Tyr178 (2.9 Å), whereas the aldehyde group was hydrogen bonded with the hydroxyl group of Thr165 (2.9 Å) (Fig. 2B). NADP^+^ was also coordinated in the cofactor-binding pocket through extensive hydrogen bonding interactions (Fig. 2B). The nicotinamide ring was stabilized by a hydrogen bond with Ser213 (3.2 Å), and the ribose oxygen formed a hydrogen bond with the amino nitrogen of Lys182 (2.7 Å). The free oxygen atom of the pyrophosphate group was hydrogen bonded with the guanidine side chain of Arg215 (3.1 Å). On the other side, the guanidine amine group of Arg41 covered the adenine ring and formed a hydrogen bond with one oxygen of the 2′-phosphate group of the ribose ring (3.0 Å). The adenosine ring was further hydrogen bonded with Asp70 (3.0 Å) and Asn138 (3.0 Å) (Fig. 2B). Site-directed mutation of Thr165, Tyr172, Arg175, Tyr178, and Lys182 to Ala residues resulted in a dramatic decrease in the overall enzymatic activity of the full-length *Rfx*MCR (Fig. 2C), confirming the essential role of these amino acid residues in malonyl-CoA reduction.

The crystal structure of *Rfx*MCR-C in complex with NADP^+^–MSA was also determined by the molecular replacement method and reﬁned to an *R*_work_ of 22.05% and an *R*_free_ of 25.28% at 2.3 Å resolution (Table 3). In contrast to *Rfx*MCR-N, both the gel filtration and AUC analyses showed the existence of a *Rfx*MCR-C monomer in solution (Fig. S5C and E). The overall structure contained 28 α-helixes and 13 β-strands that were organized into tandemly arranged SDR3 (Gly573–Glu782 and Met933–Gly962), ED (Leu783–Thr932), and SDR4 (Phe963–Val1229) domains (Fig. S3B). Superimposition of a crystal-packed *Rfx*MCR-C dimer with the full-length *Rfx*MCR yielded an RMSD of 0.995 Å (Fig. S4E and F), with the dimer interface stabilized by similar extensive hydrogen-bonding interactions as the full-length *Rfx*MCR dimer (Fig. S4B).

Similar to *Rfx*MCR-N, a cofactor NADP^+^ and intermediate MSA were resolved at the interface between the SDR3 and ED domains (Fig. 3A; Fig. S3D). In the substrate-binding pocket, MSA was immobilized by hydrogen bonding interactions with Ser731 (3.0 Å) and Tyr749 (3.2 Å) from the SDR3 domain and Arg799 (3.5 Å) from the ED. NADP^+^ was coordinated in the cofactor-binding pocket through extensive hydrogen bonding interactions with Tyr749 (2.6 Å), Lys753 (3.1 Å), Ser593 (2.7 Å), Arg616 (2.9 Å), and Asp651 (3.2 Å). The essential catalytic roles of these residues were also confirmed by enzymatic analyses (Fig. 3C). Mutation of the NADP^+^–MSA coordinating residues Ser731, Tyr749, Lys753, and Arg799 completely eliminated enzymatic activity. In addition, mutagenesis of Arg746, Arg785, and Leu795 (the highly conserved residues that are essential for CoA binding in *Pd*MCR) both markedly decreased *Rfx*MCR enzymatic activity (Fig. 3C; Fig. S7).

### *Rfx*MCR conformational changes occurred during substrate binding

**Fig 4:**
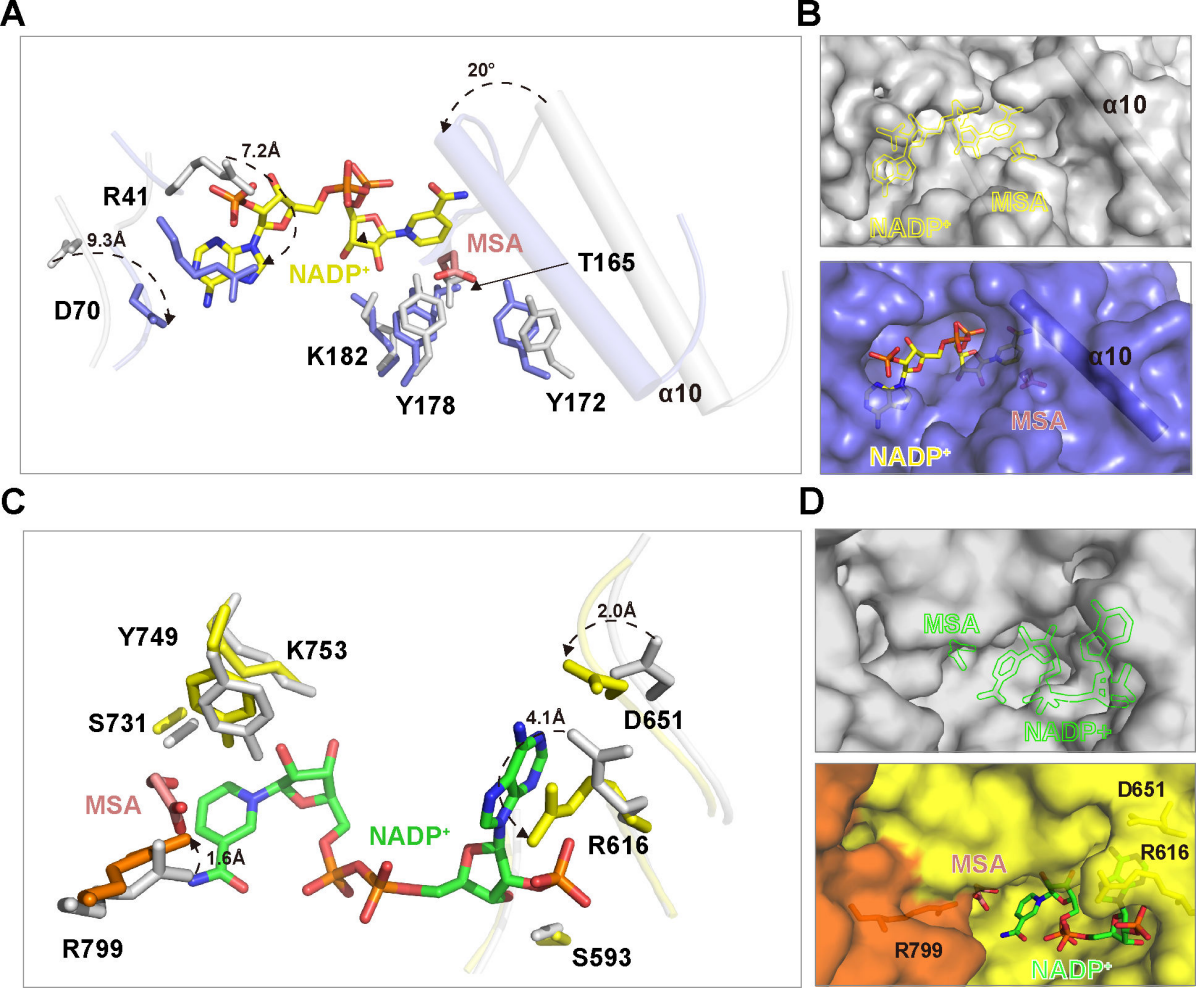
Conformational changes of the *Roseiflexus castenholzii* malonyl-CoA reductase (*Rfx*MCR) N- and C-terminal regions in response to NADP^+^–malonate semialdehyde (MSA) binding. (**A**) Superimposition of NADP^+^–MSA-bound *Rfx*MCR-N (blue) with full-length unbound *Rfx*MCR (white) revealed dramatic conformational changes in the substrate-binding pocket of the SDR1 domain. Arg41 and Asp70, which are necessary for coordinating the cofactor NADP^+^ (shown in yellow), and the catalytic triad (Thr165-Tyr178-Lys182) both underwent dramatic side-chain conformational changes. Specifically, the α10 helix (which covered MSA and the nicotinamide ring of NADP^+^) was rotated by ~20° toward the substrate-binding pocket. (**B**) Surface representation of the substrate-binding pocket in full-length unbound *Rfx*MCR (white, upper panel) and NADP^+^–MSA-bound *Rfx*MCR-N (blue, lower panel). Conformational changes of the α10 helix (shown in ribbon form) resulted in the closure of the MSA-binding site. The positions of NADP^+^ and MSA are shown as yellow outlines in full-length *Rfx*MCR. (**C**) Superimposition of NADP^+^–MSA-bound *Rfx*MCR-C (yellow) with full-length *Rfx*MCR (white) revealed striking side-chain conformational changes in the substrate-binding pocket of SDR3. Arg799, Arg616, Asp651, and the catalytic triad (Ser731–Lys753–Tyr749), which are necessary for coordinating the cofactor NADP^+^ (green) and MSA (salmon), all underwent dramatic side-chain conformational changes. (**D**) Surface representation of the substrate-binding pocket in full-length unbound *Rfx*MCR (white, upper panel) and NADP^+^–MSA-bound *Rfx*MCR-C (yellow, lower panel). The conformational changes of these key residues resulted in partial closure of the binding sites for MSA and the NADP^+^ adenosine ring. The positions of NADP^+^ and MSA are shown as green outlines in full-length *Rfx*MCR.

To investigate the conformational changes that occurred during substrate binding, we compared NADP^+^–MSA-bound *Rfx*MCR-N and *Rfx*MCR-C structures with that of full-length *Rfx*MCR (Fig. 4). Superimposition of NADP^+^–MSA-bound *Rfx*MCR-N with full-length *Rfx*MCR revealed striking conformational changes in the substrate-binding pocket. Specifically, the side chains of Arg41 and Asp70 in SDR1 were flipped by ~7.2 Å and 9.3 Å, respectively, to stabilize the NADP^+^ adenine ring. Notably, the side chains of members of the catalytic triad (Thr165–Tyr178–Lys182) were also shifted toward the substrate-binding pocket, as were the sidechains of Tyr172 and Arg175, which were essential for coordinating MSA. Especially, the α10 helix (Ser213–Gly228) was rotated by ~20° toward the substrate-binding pocket to cover MSA and the NADP^+^ nicotinamide ring (Fig. 4A). This resulted in the closure of the MSA-binding site and shrinking of the substrate-binding pocket (Fig. 4B). Interestingly, no conformational changes were observed for the corresponding helix between the apo- and NADP^+^-bound *Pd*MCR-N structures (PDB 6K8V and 6K8W), which adopted similar conformations to that of full-length *Rfx*MCR; their nicotinamide ring–binding sites were still exposed when NADP^+^ was bound (Fig. 4B; Fig. S8A and B). These analyses indicated that the dramatic conformational changes of the α10 helix were specific structural features that occurred when the reaction intermediate MSA was bound.

Similarly, extensive side chain conformational changes were observed in the *Rfx*MCR-C substrate-binding pocket during NADP^+^–MSA binding (Fig. 4C). When NADP^+^–MSA was bound, members of the SDR3 catalytic triad (Ser731–Lys753–Tyr749) were shifted toward the substrate-binding pocket to form hydrogen bonds with NADP^+^ and MSA. Specifically, the guanidine side chain of Arg799 was flipped by ~1.6 Å to stabilize the MSA carbonyl group, and the side chains of Asp651 and Arg616 were shifted by ~2.0 Å and 4.1 Å, respectively, to immobilize the adenosine ring of NADP^+^; this further resulted in partial closure of the adenosine ring–binding site in NADP^+^–MSA-bound *Rfx*MCR-C (Fig. 4D; Fig. S8C and D). In *Pd*MCR-C bound with NADP^+^ (PDB 6K8U) and CoA (PDB 6K8T), the substrate-binding pockets adopted similar conformations, even with respect to the secondary structures (Fig. S8C). There was a lack of clear electron density in the region of Glu782–Pro793 (corresponding to the α7 helix in *Pd*MCR-C); however, the nicotinamide ring–binding site was exposed in NADP^+^–MSA-bound *Rfx*MCR-C, whereas it was closed in *Pd*MCR-C bound with NADP^+^ and CoA (Fig. 4D; Fig. S8D through G), indicating that this region likely played important roles in CoA binding.

### Malonyl-CoA-binding conformations revealed by MD simulations

**Fig 5:**
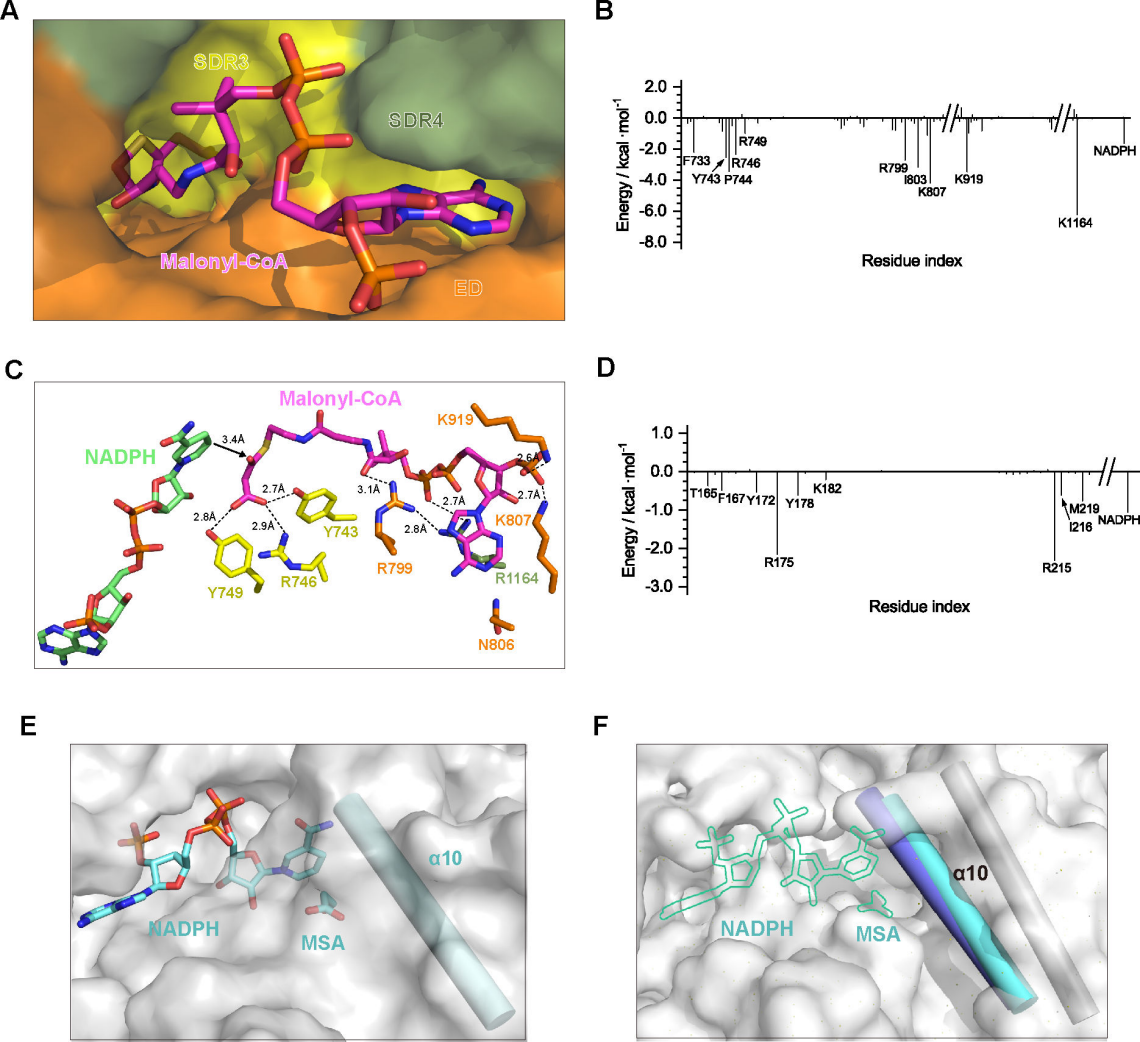
Molecular dynamics (MD) simulation and binding free-energy calculations of the full-length *Roseiflexus castenholzii* malonyl-CoA reductase (*Rfx*MCR). (**A**) Three-dimensional space-ﬁlling models of full-length *Rfx*MCR docked with the substrate malonyl-CoA (magenta). (**B**) Per-residue decomposition of binding free energy for full-length *Rfx*MCR with malonyl-CoA. Binding free energy values (kilocalories per mole) are plotted against each amino acid to show individual contributions. (**C**) Stick models of malonyl-CoA (magenta) and NADPH showing coordination in the substrate-binding pocket of full-length *Rfx*MCR. Residues necessary for malonyl-CoA binding are indicated and hydrogen-bonding interactions are shown as dashed lines. An electrostatic interaction between the carbonyl C3 atom and the hydride of the NADPH nicotinamide ring (3.4 Å) is indicated with black arrows. (**D**) Per-residue decomposition of binding free energy for full-length *Rfx*MCR with malonate semialdehyde (MSA). Binding free-energy values (kilocalories per mole) are plotted against each amino acid to show individual contributions. (**E**) Three-dimensional space-ﬁlling models of full-length *Rfx*MCR docked with the reaction intermediate MSA and cofactor NADPH (pale cyan) in the SDR1 domain. (**F**) Superimposition of NADP^+^–MSA-bound *Rfx*MCR-N (blue) with NADPH–MSA-bound full-length *Rfx*MCR (white) revealed conformational changes of the α10 helix during MSA binding.

To further explore the catalytic mechanism of *Rfx*MCR, MD simulations were performed for the substrate malonyl-CoA, the cofactor NADPH (Fig. 5A), and the reaction intermediates NADP(H)^+^–MSA with full-length *Rfx*MCR. Due to the differences in the MD starting structures and the binding cofactor, as well as the substrate-induced conformational changes, *Rfx*MCR in the binding complexes of *Rfx*MCR‒NADPH–malonyl-CoA, *Rfx*MCR‒NADP^+^–MSA, and *Rfx*MCR‒NADPH–MSA showed different RMSD profiles. But *Rfx*MCR together with the binding cofactor and substrate showed converged RMSD profiles in the second half of all MD simulations, indicating the simulation process reached the equilibration stage (Fig. S9A through C). An MM/GBSA calculation (47) revealed that the highest binding free energy of malonyl-CoA to full-length *Rfx*MCR was −77.76 kcal/mol (Table 4), indicating that malonyl-CoA was a much more favorable substrate than MSA for binding to *Rfx*MCR. The binding free energy was further decomposed to identify per-residue contributions (Fig. 5B). The largest per-residue contribution came from Arg1164 in the SDR4 domain, which formed hydrogen bonds with the pyrophosphate oxygen of CoA and the guanidine nitrogen of Arg799 (Fig. 5B and C). Similarly, the residues Arg799, Lys807, and Lys919 (from the ED domain) contributed to immobilize the CoA moiety through hydrogen bonding interactions (Fig. 5C). The essential roles of these amino acid residues in malonyl-CoA binding were further verified by the decreased binding free energy of Arg799 and the absence of Lys807 and Lys919 in the per-residue decomposition of NADPH–MSA-bound *Rfx*MCR (Fig. S9D). These results were also in agreement with the structural comparisons of NADP^+^–MSA-bound *Rfx*MCR-C and full-length *Rfx*MCR, in which Arg799 underwent dramatic conformational changes during MSA binding (Fig. 4C).

**TABLE 4:** Binding free energies calculated using the MM/GBSA approach

Binding complex	Energy components^*a*^*^,b^*				
	Δ*E*_ele_	Δ*E*_vdW_	Δ*G*_GB_	Δ*G*_SA_	Δ*G*_bind^*b*^_
*Rfx*MCR‒M-CoA	253.70 ± 9.48	−60.91 ± 4.60	−260.58 ± 8.57	−9.97 ± 0.45	−77.76 ± 4.40
*Rfx*MCR(N)^*c*^‒MSA	42.57 ± 2.31	−7.35 ± 3.21	−50.14 ± 1.77	−1.83 ± 0.52	−16.75 ± 2.54
*Rfx*MCR(C)^*d*^‒MSA	34.69 ± 4.25	−7.36 ± 2.64	−36.86 ± 3.50	−2.17 ± 0.59	−11.69 ± 2.14

^*a*^ Energies are in kilocalories per mole.

^*b*^ Δ*G*_bind_ ≈ Δ*E*_ele_ + Δ*E*_vdW_ + Δ*G*_GB_ + Δ*G*_SA._

^*c*^ *Rfx*MCR(N) represents that the NADP^+^–MSA-bound at the N-terminal SDR1–2 domains of full-length *Rfx*MCR.

^*d*^ *Rfx*MCR(C) represents that the NADPH–MSA-bound at the C-terminal SDR3–4 domains of full-length *Rfx*MCR.

Importantly, the malonate moiety of malonyl-CoA was hydrogen bonded with residues Tyr749, Arg746, and Tyr743 within the sequence motif YXXRXXY that were conserved between SDR3 and SDR1 domains (Fig. 5C; Fig. S6C). In contrast with Arg746 and Tyr749, which contributed to both malonyl-CoA and MSA binding, Tyr743 made significant contributions to malonate binding but no contributions to MSA binding (Fig. 5B through D; Fig. S9D). This indicated that Ty743 played an essential role in malonyl-CoA reduction. MD simulations of the reduction intermediate NADP(H)–MSA with full-length *Rfx*MCR revealed a relatively higher binding affinity with the SDR1–2 domains than with SDR3–ED–SDR4 (Table 4), demonstrating that it was more favorable for MSA to bind to the SDR1–2 domains to proceed through the second reduction step of MSA to 3-HP. Consistent with the crystal structure of NADP^+^–MSA-bound *Rfx*MCR-N, Thr165, Tyr172, Arg175, and Tyr178 contributed considerable free energies for MSA binding to full-length *Rfx*MCR. Specifically, Arg175 and Arg215 contributed the biggest free energies for MSA binding (Fig. 5D; Fig. 2B). Coincidently, the amino acid residues Arg215, Ile216, and Met219, which had moderate binding free energies, were all located within the α10 helix (Ser213–Gly228) that underwent striking conformational changes during MSA binding (Fig. 5D; Fig. 4A). Superimposition analyses revealed that, in the simulated NADPH–MSA-bound full-length *Rfx*MCR, the α10 helix also adopted the same conformations that were observed in the crystal structure of NADP^+^–MSA-bound *Rfx*MCR-N (Fig. 5E and F). This confirmed that the conformational changes of the α10 helix were necessary for MSA binding and closure of the binding pocket for subsequent reduction reactions.

### Molecular mechanism of *Rfx*MCR in catalyzing the two-step reduction of malonyl-CoA to 3-HP

**Fig 6:**
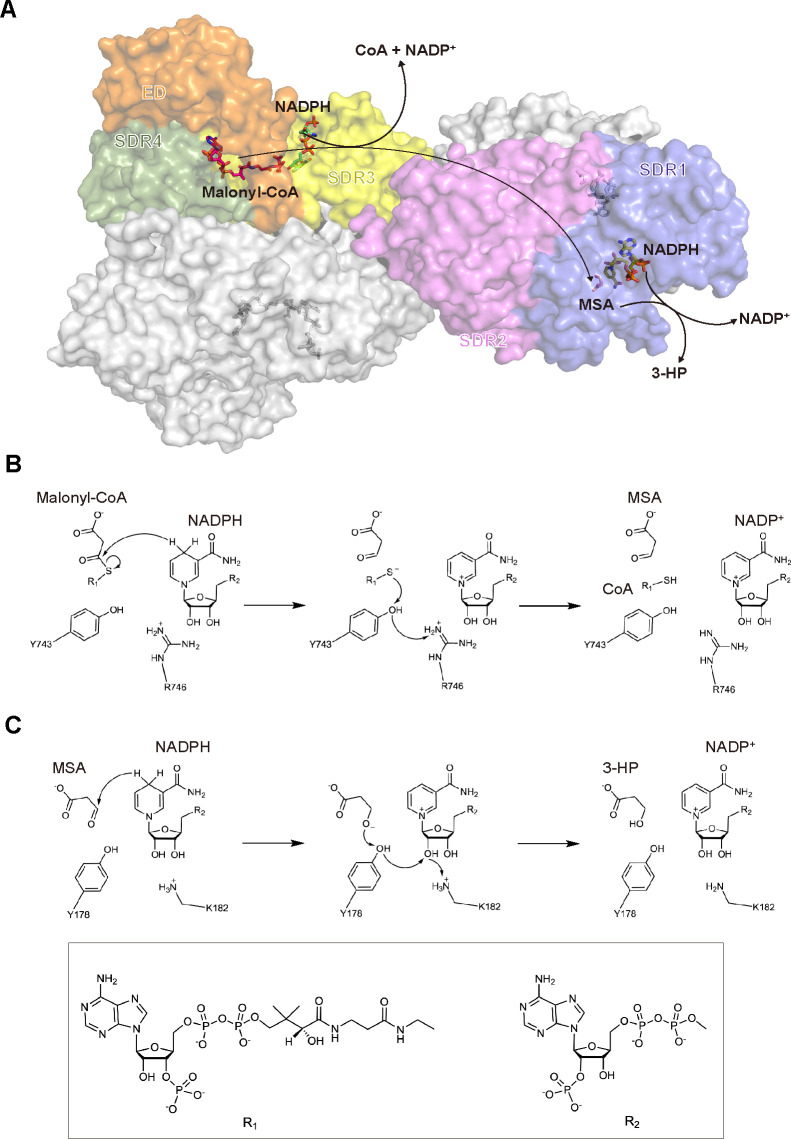
Proposed catalytic mechanism of *Roseiflexus castenholzii* malonyl-CoA reductase (*Rfx*MCR) as a bi-functional MCR. (**A**) Diagram of the two-step reduction reactions catalyzed by full-length *Rfx*MCR. The transport pathways of malonyl-CoA (magenta), the cofactor NADPH (green), and the reaction intermediate malonate semialdehyde (MSA, salmon) are shown in one subunit of the homodimer, as indicated by the crystal structure data and the molecular dynamics simulation models. One of the two subunits is colored in gray, and the short-chain dehydrogenase/reductase (SDR) 1, SDR2, SDR3, and SDR4 domains and the extra domain (ED) of the other subunit are colored in blue, violet, yellow, sage, and orange, respectively. (**B, C**) The proposed mechanism by which *Rfx*MCR catalyzes a two-step reduction of malonyl-CoA to MSA and further reduction of MSA to 3-hydroxypropanoate (3-HP). The functional groups of malonyl-CoA and NADPH are indicated as R_1_ and R_2_, respectively.

In the equilibrated binding structure with NADPH–malonyl-CoA, electrostatic interactions were observed between the carbonyl C3 atom and the hydride from the NADPH nicotinamide ring (3.4 Å); this provided the prerequisite for a hydrophilic attack at the C3 atom. On the other side of the molecule, oxygen atoms at the malonyl end were immobilized in a chain of hydrogen bonding interactions with the OH of Tyr743 (2.7 Å), the NH1 of Arg746 (2.9 Å), and Tyr749 (2.8 Å) (Fig. 5C). Based on the crystal structures of NADP^+^–MSA-bound *Rfx*MCR-N and *Rfx*MCR-C, and the binding characteristics of full-length *Rfx*MCR with NADPH–malonyl-CoA and NADP(H)^+^–MSA, we propose a catalytic mechanism for the two consecutive reduction reactions (Fig. 6A). Initially, the hydride from the NADPH nicotinamide group launches a nucleophilic attack at the carbonyl C3 atom of malonyl-CoA, leading to breakage of the S‒C bond and generation of MSA, a CoAS^−^ thioanion, and NADP^+^ (Fig. 6B). Subsequently, CoAS^−^ is reduced by sequential deprotonation of the Tyr743 hydroxyl group and the Arg746 guanidine amine; the deprotonated Arg746 could be restored by surrounding solvents. As indicated in the structural analyses of NADP^+^–MSA-bound *Rfx*MCR-C, the reaction intermediate MSA could be further stabilized in a proton relay network by hydrogen bonding interactions with Ser731, Tyr749, and Arg799 for the second reduction step (Fig. 3B).

In the structure of NADP^+^–MSA-bound *Rfx*MCR-N, Thr165 stabilized the reaction intermediate MSA by forming hydrogen bonds with the C3 carbonyl oxygen (Fig. 2B), which facilitates nucleophilic attack by the NADPH hydride on the C3 atom of MSA. The MSA anion generated by this process is protonated by extracting a proton from the Tyr178 hydroxyl group to form 3-HP (Fig. 6C). Due to the steric hindrance and the distance between the Tyr178 hydroxyl and the Lys182 α-amino group (4.6 Å in the crystal structure) (Fig. 2B), a direct proton transfer from Lys182 to Tyr178 is unlikely. However, Lys182 could play a dual role not only in orienting the cofactor NADPH by hydrogen bonding with the nicotinamide ribose (2.7 Å) but also by mediating protonation of Tyr178 through an NADPH hydroxyl-mediated proton transfer (Fig. 2B; Fig. 6C). The deprotonated Lys182 could feasibly be recovered by the solvent, completing the catalytic reaction. Overall, using two molecules of NADPH, malonyl-CoA could be converted into MSA, then further reduced to 3-HP through two consecutive reactions occurring at domains SDR3–ED–SDR4 and SDR1–2 of *Rfx*MCR (Fig. 6).

## DISCUSSION

We here investigated the catalytic mechanism of a bi-functional MCR from *R. castenholzii*. It is a chlorosome-less green nonsulfur bacterium that is closely related to *C. aurantiacus* (20), the species in which the 3-HP autotrophic carbon fixation pathway was first identified and demonstrated. For the first time, we determined the cryo-EM structure of a full-length bi-functional MCR and found that it contained two subunits, each with four tandemly arranged SDR domains and one ED domain, which were cross-interlocked to form a homodimer. By determining the cryo-EM structure of full-length *Rfx*MCR, we first observed that the *Rfx*MCR-N and *Rfx*MCR-C in each subunit were connected by an α-helix (G^573^WAESL^578^). Second, we revealed a homodimer of two cross-interlocked subunits with the N- and C-terminal ends in the same direction. In the center of the homodimer, two of these helices were antiparallelly arranged and serve as a point of intersection of the two subunits, further representing that the two subunits were interlocked but not paring contacted to form the homodimer (Fig. 1E). This disagrees with the previous studies of *Pd*MCR. In this work, the authors fit the dimeric crystal structures of *Pd*MCR-N and *Pd*MCR-C into the SAXS model of full-length *Pd*MCR and proposed that two *Pd*MCR subunits were paring contacted to form a homodimer (19).

MSA is an essential reaction intermediate of the malonyl-CoA reduction, and it is either released or remains enzyme bound (17). Like other aldehydes, cellular accumulated MSA can react with free amino groups to form adducts and exert toxic effects. The growth of *E. coli* K-12 cells at high temperature was severely restricted by the accumulation of MSA when pyrimidines were used as the sole nitrogen source (5). The bi-functional MCR is superior to the mono-functional enzymes in that it combines the alcohol and aldehyde dehydrogenase (CoA-acylating) activities into one single enzyme, which directly consumes the reaction intermediate MSA and thus decreases its cellular accumulation and toxicity. However, no structural information of the MSA-bound conformation of MCR enzymes is available. Notably, we here determined the crystal structures of NADP^+^–MSA-bound *Rfx*MCR-N and *Rfx*MCR-C and revealed the conformational changes necessary for substrate selection and intermediate coordination. Superimposition of the catalytic SDR1 domain with the non-catalytic SDR2 domain gave a main chain RMSD of 6.680 Å. Compared to SDR1, the non-catalytic SDR2 lacked the α10 and α11 helices, which were necessary for covering the NADP^+^–MSA binding pocket; it also lacked two antiparallel β-strands (β7–β8) and two short α-helices (α5–α6) that were exposed on the SDR1 surface (Fig. S6A). Consistent with the crystal structure of NADP^+^–MSA-bound *Rfx*MCR-N, MD simulations of the full-length *Rfx*MCR with NADP(H)^+^–MSA revealed similar conformational changes of the α10 helix, confirming that this helix played an important role in stabilizing the binding of the reaction intermediate MSA (Fig. 4A; Fig. 5F). In addition, SDR2 and SDR1 had completely different amino acid residues in the NADP^+^–MSA binding sites; SDR2 had a distinct substrate-binding pocket that disfavored MSA binding (Fig. S6A; Table 4). Similarly, the superimposition of the SDR3 and SDR4 main chains had a larger RMSD at 18.511 Å (Fig. S6B), indicating the dramatic differences between these two SDR domains arose from the incorporation of ED into the SDR3 domain. Sequence alignment further confirmed that the substrate-binding residues in SDR3 and SDR4 were less conserved, which resulted in the absence of a malonyl-CoA–NADPH binding pocket in SDR4 (Fig. S6B).

Interestingly, the superimposition of *Rfx*MCR-N and *Rfx*MCR-C revealed an excellent match of the tandem SDR1–2 and SDR3–4 domain core architectures and relatively high conservation of the NADP^+^–MSA binding site (Fig. S6C). Notably, SDR1 required additional secondary structures (such as the α10 and α11 helices) to cover the MSA-binding site. In contrast, SDR3 incorporated a larger module, including SDR4 and ED, to form the active site pocket, in which Arg1164 and Arg799 made major contributions to malonyl-CoA binding (Fig. 5C; Fig. S6C; Table 4). As a result, the conformation of the substrate-binding pocket in SDR3 was more favorable for binding the substrate malonyl-CoA, whereas the SDR1-binding pocket was optimal for stabilizing the reaction intermediate MSA. However, the mutation of the amino acid residues in SDR4 of *Cfx*MCR increases enzymatic activity (18). Therefore, although SDR2 and SDR4 were not involved in either substrate or cofactor binding or catalysis, they served as auxiliary domains to maintain *Rfx*MCR catalytic activity. Most importantly, we found that the substrate specificity of the bi-functional MCR was determined by incorporating additional secondary structures to the core SDR architectures (Fig. S6).

*Rfx*MCR has not been examined in biosynthetic 3-HP production, but *Cfx*MCR has been reconstructed in *E. coli* BL21 (DE3) cells together with *Cfx*ACCase to generate a 3HP-producing strain (9). Separation of the *Cfx*MCR-N and *Cfx*MCR-C fragments in this strain increases 3-HP production by 1.5 times (17), indicating that the full-length *Cfx*MCR enzyme activity is regulated by coordination between the *Cfx*MCR-N and *Cfx*MCR-C modules. However, the expression levels of *Cfx*MCR-N and *Cfx*MCR-C in the cells were different, which generated a functional imbalance that further decreased the 3-HP yield. Direct evolution of *Cfx*MCR-C has yielded three mutants with increased enzymatic activity (Fig. S10A) and 270-fold higher 3-HP production (after fine-tuning of *Cfx*MCR-N expression levels) (17, 18); these mutants were N940V (corresponding to Asn951 in *Rfx*MCR), K1106W (Lys1116 in *Rfx*MCR), and S1114R (Asn1124 in *Rfx*MCR). The highly conserved Lys1116 was located at the interface between SDR4 and the ED domain. Mutation of Lys1116 to Trp would increase steric hindrance with Asn887 from the helix α33, which likely enhanced malonyl-CoA binding by facilitating closure of the substrate-binding pocket during catalysis (Fig. S10B). Mutation of the conserved Asn951 to Val likely enhanced the hydrophobic interactions with residues Pro934 and Pro959 located in the α36 and loop region covering the substrate-binding pocket, which probably enhanced NADPH binding during catalysis (Fig. S10C). In contrast, Asn1124, which was located at the end of the SDR4 domain, was not conserved and was far away from the substrate pocket (Fig. S10D). Combination of these mutations enhanced the enzyme activity of *Cfx*MCR-C, but the mutation “hot spots” identified via saturation mutation were distant from the substrate and cofactor-binding pockets. Nevertheless, the structural basis of *Rfx*MCR revealed in this work serves as an accurate template for the future rational design of the full-length MCR enzymes, which contributes to eliminate the expression and functional imbalance between dissected MCR-N/C and also decreases the cytotoxicity resulting from the accumulation of the toxic reaction intermediate MSA.

In summary, we here revealed the molecular bases underlying substrate binding, specificity determination, and catalytic mechanisms of a bi-functional MCR from *R. castenholzii*. This was achieved through a combination of approaches, including cryo-EM, X-ray crystallography, enzymatic analyses, and MD simulations. The results of this study not only broaden our understanding of the key catalytic steps in the 3-HP autotrophic carbon fixation pathways but are expected to advance industrial applications of this metabolic pathway in biosynthetic 3-HP production.

## Data Availability

Cryo-EM maps and atomic coordinates of full-length *Rfx*MCR have been deposited into the Electron Microscopy Data Bank (accession codes EMD-34812) and the Protein Data Bank (PDB) (accession codes 8HI4), respectively. The structure factors and coordinates of NADP^+^–MSA-bound *Rfx*MCR-N and *Rfx*MCR-C have been deposited in the Protein Data Bank under the accession codes 8HI6 and 8HI5. Other data are available from the corresponding authors on reasonable request.

## AFTER EPUB

*[This article was published on 6 June 2023 with a typographical error in Materials and Methods. The error was corrected in the current version, posted on 29 August 2023.]*

## SUPPLEMENTAL MATERIAL

Fig. S1 mbio.03233-22-s0001.tif Purification and cryo-EM analysis of the full-length malonyl-CoA reductase (*Rfx*MCR) from *R. castenholzii*. (A-C) SDS-PAGE (A), gel filtration (B) and Native PAGE (C) of the full-length *Rfx*MCR. (D) Representative raw particles from an original cryo-EM micrograph of the full-length *Rfx*MCR. (E) Representative reference-free 2D class averages of the full-length *Rfx*MCR. (F) Gold-standard Fourier shell correlation (FSC=0.143) curves of the *Rfx*MCR. The no mask (blue), loose (green), tight (red) and corrected (purple) curves are shown with different colors. (G) Local resolution of the cryo-EM map estimated by ResMap. (H) Gel filtration of wild type (WT), single mutant (E576A, S), double mutant (W574AE576A, D) and triple mutant (W574AE576AF579A, T) *Rfx*MCR. (I) Native PAGE (left) and SDS-PAGE (right) of wild type (WT), single mutant (E576A, S), double mutant (W574AE576A, D) and triple mutant (W574AE576AF579A, T) *Rfx*MCR.

Fig. S2 mbio.03233-22-s0002.tif Flowchart of the cryo-EM data processing for the full-length *Rfx*MCR. To generate a template for two-dimensional (2D) classification, 14,112 particles were auto-picked from 20 micrographs, and subjected to 2D classification. Using a 2D reference generated from 12,105 particles, 4,043,667 particles were auto-picked from 4,647 micrographs and imported in CryoSPARC. After five iterative rounds of 2D classifications, 2,391,743 particles were selected and imported in RELION for generating a 3D map with the initial model program, which was used as a reference for three-dimensional (3D) classifications that generated four classes of particles. Then 1,766,605 particles from the best two classes (percentage of 42.89% and 30.97%, respectively) were selected and extracted, and subjected to another round of reference-based 3D classiﬁcation and non-uniform reﬁnement in CryoSPARC, which produced an EM-map with a global resolution of 3.35 Å based on the gold standard Fourier shell correlation (FSC).

Fig. S3 mbio.03233-22-s0003.tif Fit of the *R. castenholzii* malonyl-CoA reductase (*Rfx*MCR), cofactor NADP^+^ and reaction intermediate MSA in the representative densities. (A) Fit of the full-length *Rfx*MCR model in the representative cryo-EM densities. (B) Secondary structure topology of the full-length *Rfx*MCR. (C and D) The density maps for the cofactor NADP^+^ and reaction intermediate MSA bound in the crystal structures of *Rfx*MCR-N (C) and *Rfx*MCR-C (D). The electron density maps are contoured as *2mFo-DFc* at 2.0 σ level in gray. (E-F) The cryo-EM map (contoured at 1.5σ) of the substrate binding pocket of full-length *Rfx*MCR incubated with NADP^+^ (E, 4.0 Å resolution) or malonyl-CoA (F, 4.2 Å resolution). The positions of NADP^+^ and malonyl-CoA are shown as green and magenta outlines to show the lack of cryo-EM densities.

Fig. S4 mbio.03233-22-s0004.tif Crystal packing and dimer interface of the NADP^+^–MSA-bound *Rfx*MCR-N and *Rfx*MCR-C, and the superimpositions with full-length *Rfx*MCR. (A and B) The N- (A) and C-terminal (B) dimer interface of the full-length *Rfx*MCR. The amino acid residues involved in forming hydrogen bonding interactions are shown in stick models, the hydrogen bonds are shown in dashed lines and labeled with distances. One subunit in the homodimer is colored in gray. (C and E) Crystal packing of the NADP^+^–MSA-bound *Rfx*MCR-N (C) and *Rfx*MCR-C (E). A homodimer (indicated with dashed line) was identified in the crystal packing. (D and F) Superimpositions of the NADP^+^–MSA-bound *Rfx*MCR-N (D) and *Rfx*MCR-C (F) with the full-length *Rfx*MCR (gray). The SDR1, SDR2, SDR3, SDR4 and ED domains are colored in blue, violet, yellow, sage and orange, respectively. The bound NADP^+^ (yellow in SDR1 and green in SDR3) and MSA (salmon) are shown in stick models.

Fig. S5 mbio.03233-22-s0005.tif Purification and analytic ultracentrifugation (AUC) of the *Rfx*MCR-N and *Rfx*MCR-C. (A-C) Gel filtration analyses (A and C) and SDS-PAGE (B) of the *Rfx*MCR-N (A) and *Rfx*MCR-C (C). The absorption of the *Rfx*MCR-N (A) and *Rfx*MCR-C (C) at 280 nm against the elution volume (mL) were recorded. (D-E) Sedimentation velocity analytical ultracentrifugation (AUC) of the *Rfx*MCR-N (D) and *Rfx*MCR-C (E). The upper panel shows the raw data from the time-course measurement of absorbance of the sample at 280 nm along the sample cell length. The middle panel shows residuals after fitting the data to the continuous size-distribution model. The lower panel shows the continuous mass distribution for the calculated solution. The curve indicates that the *Rfx*MCR-N (D) exists as a homodimer in solution (the calculated MW of the monomer is 63.4 kDa), and the *Rfx*MCR-C (E) exists as a monomer in solution (the calculated MW of the monomer is 72.9 kDa).

Fig. S6 mbio.03233-22-s0006.tif Structural comparisons of the catalytic and non-catalytic SDR domains in *Rfx*MCR. (A) Superimposition of the NADP^+^–MSA-bound SDR1 domain (blue) with the non-catalytic SDR2 domain (violet) gives a RMSD at 6.680 Å. The NADP^+^ (yellow) and MSA (salmon) are shown in stick models. The NADP^+^–MSA-bound SDR1 (right top) contains typical substrate binding pocket, whereas the non-catalytic SDR2 domain (right bottom) does not contain an optimal pocket for substrate binding. Sequence alignment of the SDR1 and SDR2 showed that the SDR2 does not contain conserved amino acid residues for substrate binding. The amino acid residues for substrate binding and catalysis are indicated with red stars. (B) Superimposition of the NADP^+^–MSA-bound SDR3 domain (yellow for SDR3 and orange for the ED) with the non-catalytic SDR4 domain (sage) gives a RMSD at 18.511 Å. The SDR3 (right top) but not SDR4 domain (right bottom) contains typical substrate binding pocket for NADP^+^–MSA binding. Sequence alignment of the SDR3 and SDR4 showed that the SDR4 does not contain conserved amino acid residues for substrate binding. The amino acid residues for substrate binding and catalysis in SDR3 are indicated by blue stars. (C) Superimposition of the NADP^+^–MSA-bound *Rfx*MCR-N and *Rfx*MCR-C structures. Surface presentation of *Rfx*MCR-N (right top) and *Rfx*MCR-C (right bottom) indicated distinct conformations of the substrate binding pockets. Sequence alignment of the *Rfx*MCR-N and *Rfx*MCR-C showed that the catalytic SDR1 and SDR3 domains contain strictly conserved amino acid residues for substrate binding and catalysis.

Fig. S7 mbio.03233-22-s0007.tif Structure-based sequence alignment of the bi-functional MCRs from *Roseiflexus castenholzii* (*Rfx*MCR, WP_012121415.1), *Chloroflexus aurantiacus* (*Cfx*MCR, WP_012258473.1) and *Porphyrobacter dokdonensis* (*Pd*MCR, WP_068862557.1). The secondary structures of *Rfx*MCR are indicated on top of the sequence. The catalytic and substrate binding residues of *Rfx*MCR-N and *Rfx*MCR-C are indicated by red and blue stars, respectively. The YXXRXXY motifs are present in both the catalytic and substrate-binding sites of *Rfx*MCR-N (Y^172^YGRIAY^178^) and *Rfx*MCR-C (Y^743^PNRSDY^749^), in which the underlined letters represent the non-conserved amino acid residues.

Fig. S8 mbio.03233-22-s0008.tif Conformational changes of *Rfx*MCR-N and *Rfx*MCR-C in binding with NADP^+^–MSA. (A) Superimposition of the substrate binding pocket of NADP^+^–MSA-bound *Rfx*MCR-N (blue) with that of full-length *Rfx*MCR (gray), apo- (green) and NADP^+^-bound (wheat) *Pd*MCR-N. (B) The substrate binding pocket of the NADP^+^-bound *Pd*MCR-N is shown in surface. The NADP^+^ is shown in stick models. (C) Superimposition of the substrate binding pockets of NADP^+^–MSA-bound *Rfx*MCR-C (yellow) with that of full-length *Rfx*MCR (gray), the NADP^+^-bound (wheat) and CoA-bound (pale green) *Pd*MCR-C. The α33 helix covering the CoA binding site is shown in ribbon, and the NADP^+^, MSA and CoA are shown in stick models. (D) Surface presentation of the substrate binding pocket of full-length *Rfx*MCR (gray), the positions for binding the CoA (cyan), NADP^+^ and MSA (yellow) are shown and outlined. (E) Surface presentation of the substrate binding pocket of NADP^+^–MSA-bound *Rfx*MCR-C (yellow). (F and G) Surface presentation of the substrate binding pocket of the NADP^+^-bound (F, wheat) and CoA-bound (G, pale green) *Pd*MCR-C.

Fig. S9 mbio.03233-22-s0009.tif Molecular dynamics (MD) simulations of the full-length *Rfx*MCR. (A-C) MD simulations of the full-length *Rfx*MCR in complex with malonyl-CoA and NADPH (A), NADP^+^-MSA (B) and NADPH-MSA (C). (A) Root-mean-square deviations (RMSDs) of full-length *Rfx*MCR (black), the bound cofactor NADPH (green), and substrate malonyl-CoA (magenta) during MD simulations were plotted against the simulation time (ns). (B) RMSDs of full-length *Rfx*MCR (black), the bound cofactor NADP^+^ (yellow), and intermediate MSA (salmon) during MD simulations were plotted against the simulation time (ns). (C) RMSDs of full-length *Rfx*MCR (black), the bound cofactor NADPH (green), and intermediate MSA (salmon) during MD simulations were plotted against the simulation time (ns). (D) The per-residue decomposition of the binding free energies of full-length *Rfx*MCR in binding with MSA and NADP^+^.

Fig. S10 mbio.03233-22-s0010.tif Spatial organization of *Rfx*MCR amino acid residues that are corresponding to the hot spot mutations of *Chloroflexus aurantiacus* MCR (*Cfx*MCR). (A) Distribution of the amino acid residues that are corresponding to the hot spot mutations in *Cfx*MCR. The catalytic centers of *Rfx*MCR are indicated with bound substrate malonyl-CoA (magenta) and cofactor NADPH (green), the SDR3 (yellow), SDR4 (sage) and ED (orange) domains are shown in surface, and the amino acid residues are shown in stick models. (B-D) Spatial organizations of the amino acid residues Lys1116 (B), Asn951 (C) and Asn1124 (D) that are corresponding to the hot spot mutations K1106W, N940V and S1114R of *Cfx*MCR. The amino acid residues in *Rfx*MCR are shown in stick models, and the mutations in *Cfx*MCR are shown as gray outline.

ASM does not own the copyrights to Supplemental Material that may be linked to, or accessed through, an article. The authors have granted ASM a non-exclusive, world-wide license to publish the Supplemental Material files. Please contact the corresponding author directly for reuse.
