# Supplementary figures and images for: Structural basis of a bi-functional malonyl-CoA reductase (MCR) from the photosynthetic green non-sulfur bacterium Roseiflexus castenholzii
Source: mBio. 2023 Jun 6;14(4):e03233-22. doi: 10.1128/mbio.03233-22 (PMC10470521; doi:10.1128/mbio.03233-22)

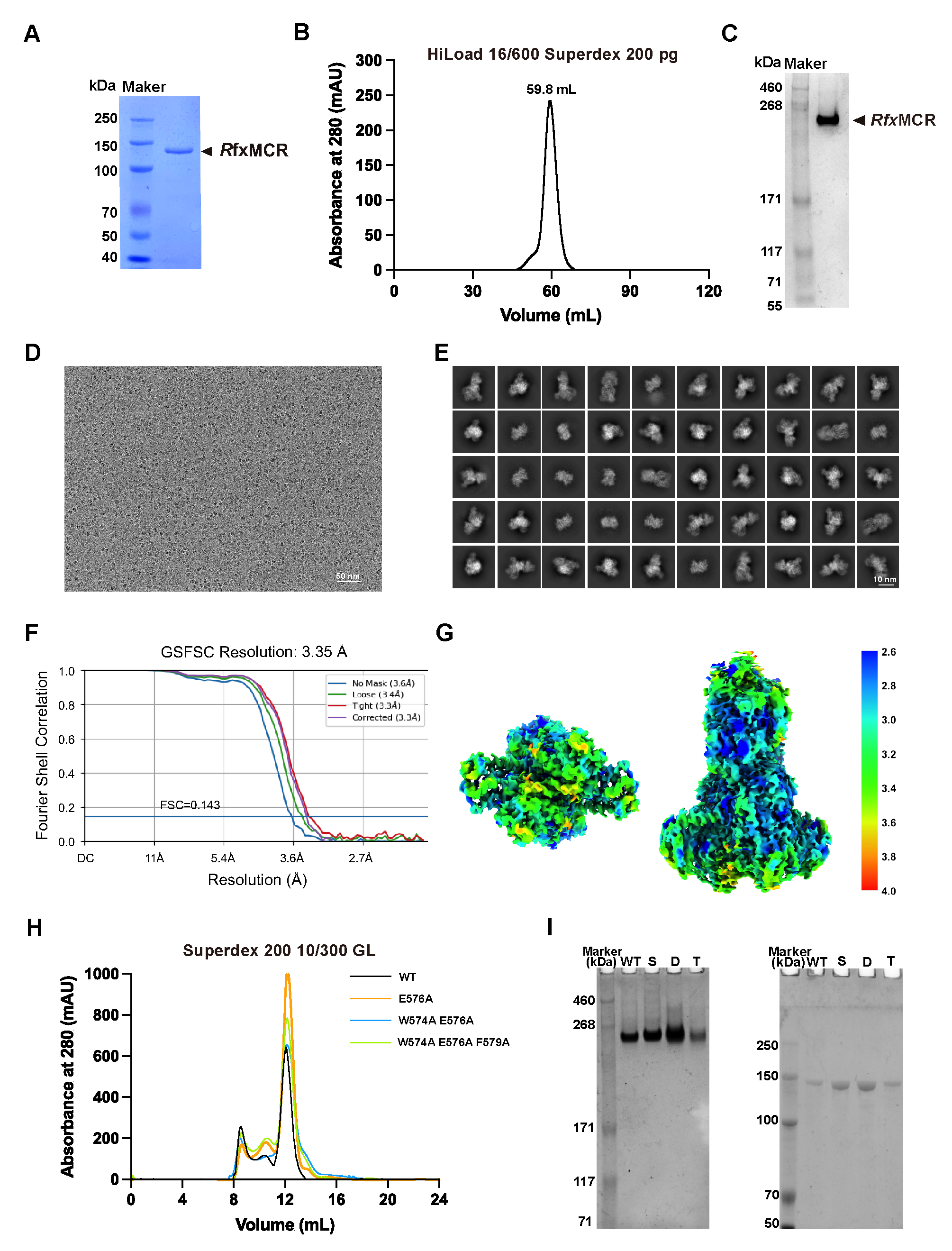

Supplement: Fig. S1 — Purification and cryo-EM analysis of the full-length malonyl-CoA reductase (RfxMCR) from R. castenholzii. (A-C) SDS-PAGE (A), gel filtration (B) and Native PAGE (C) of the full-length RfxMCR. (D) Representative raw particles from an original cryo-EM micrograph of the full-length RfxMCR. (E) Representative reference-free 2D class averages of the full-length RfxMCR. (F) Gold-standard Fourier shell correlation (FSC=0.143) curves of the RfxMCR. The no mask (blue), loose (green), tight (red) and corrected (purple) curves are shown with different colors. (G) Local resolution of the cryo-EM map estimated by ResMap. (H) Gel filtration of wild type (WT), single mutant (E576A, S), double mutant (W574AE576A, D) and triple mutant (W574AE576AF579A, T) RfxMCR. (I) Native PAGE (left) and SDS-PAGE (right) of wild type (WT), single mutant (E576A, S), double mutant (W574AE576A, D) and triple mutant (W574AE576AF579A, T) RfxMCR. [file mbio.03233-22-s0001.tif]

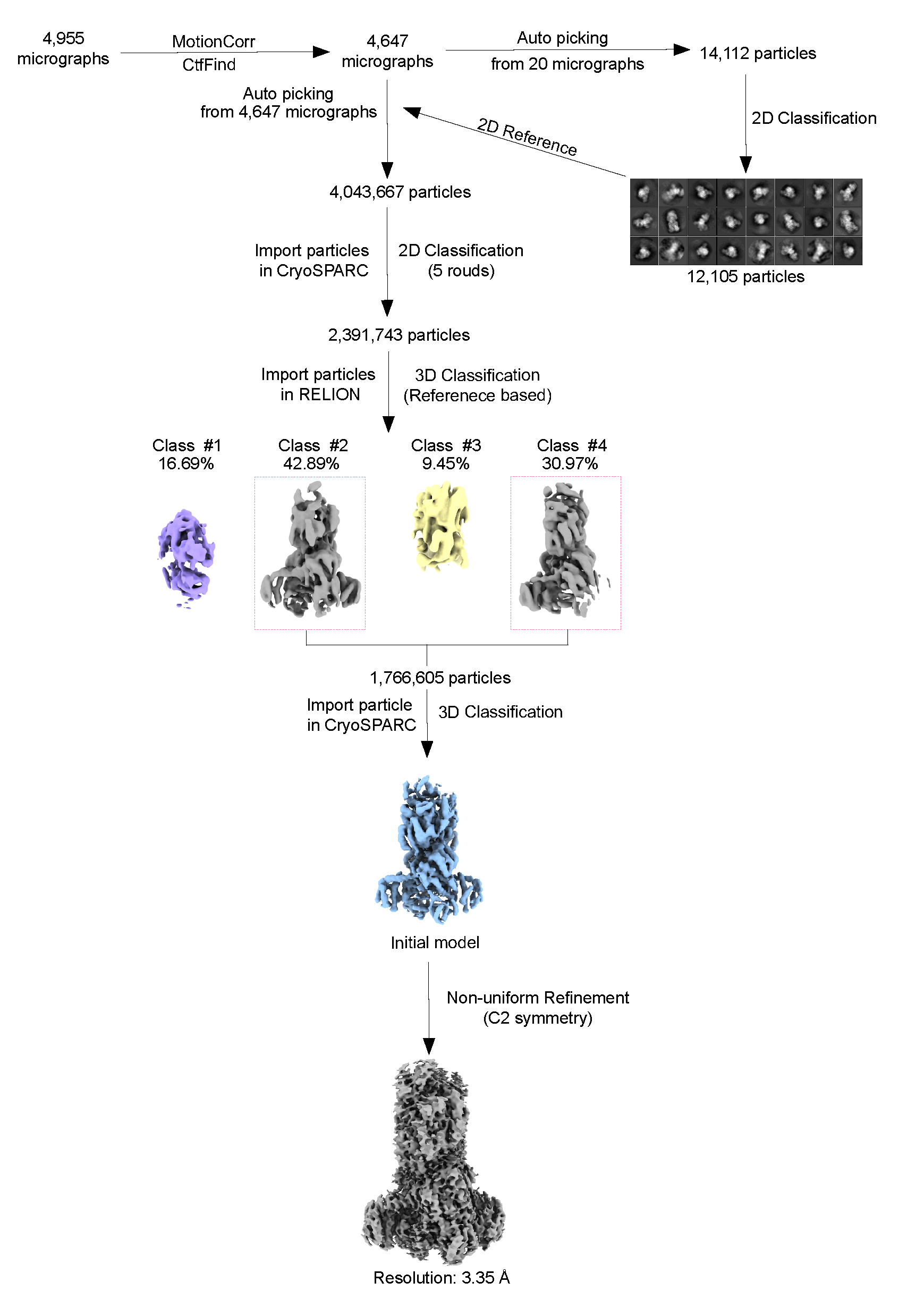

Supplement: Fig. S2 — Flowchart of the cryo-EM data processing for the full-length RfxMCR. To generate a template for two-dimensional (2D) classification, 14,112 particles were auto-picked from 20 micrographs, and subjected to 2D classification. Using a 2D reference generated from 12,105 particles, 4,043,667 particles were auto-picked from 4,647 micrographs and imported in CryoSPARC. After five iterative rounds of 2D classifications, 2,391,743 particles were selected and imported in RELION for generating a 3D map with the initial model program, which was used as a reference for three-dimensional (3D) classifications that generated four classes of particles. Then 1,766,605 particles from the best two classes (percentage of 42.89% and 30.97%, respectively) were selected and extracted, and subjected to another round of reference-based 3D classiﬁcation and non-uniform reﬁnement in CryoSPARC, which produced an EM-map with a global resolution of 3.35 Å based on the gold standard Fourier shell correlation (FSC). [file mbio.03233-22-s0002.tif]

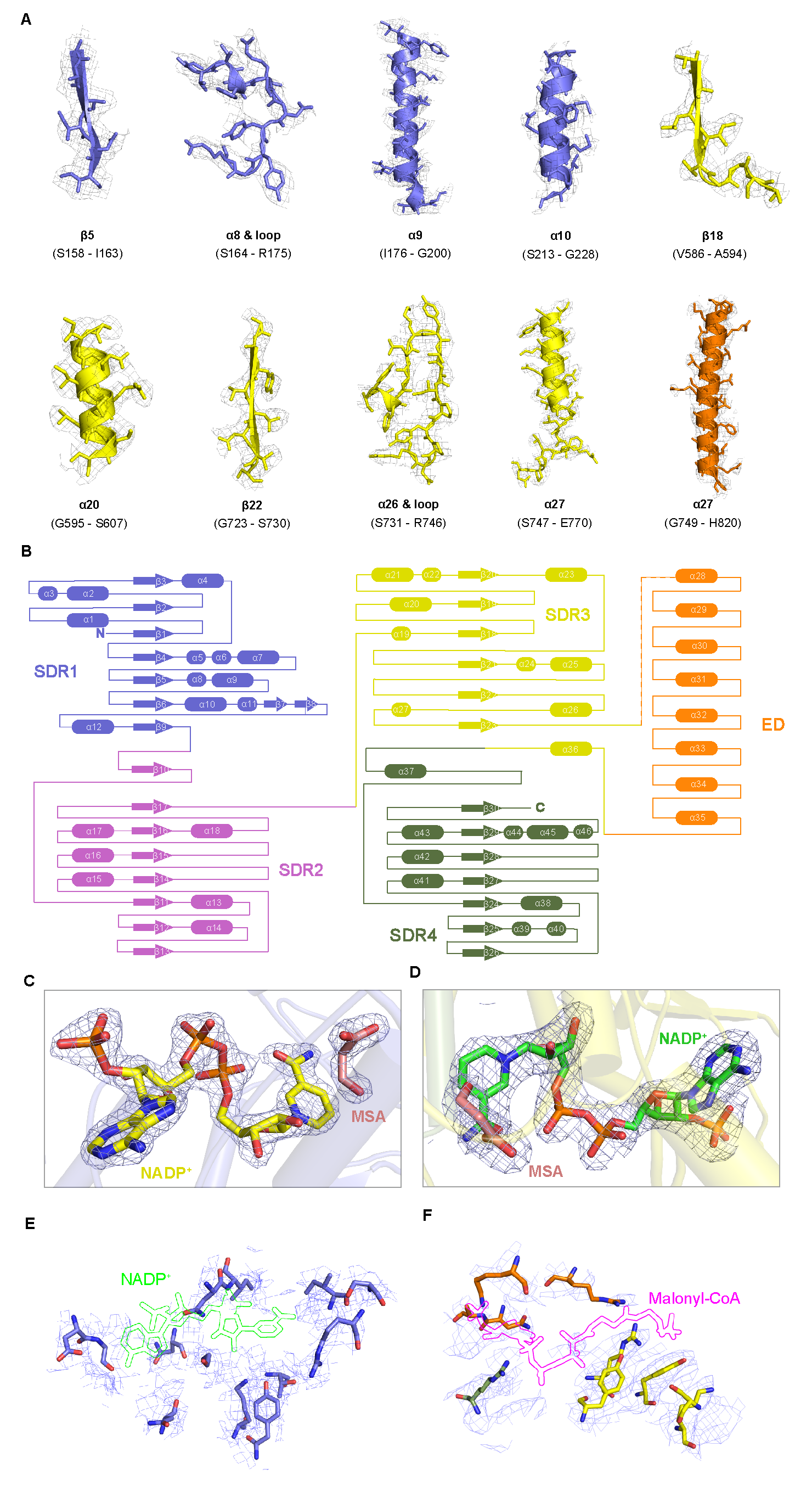

Supplement: Fig. S3 — Fit of the R. castenholzii malonyl-CoA reductase (RfxMCR), cofactor NADP+ and reaction intermediate MSA in the representative densities. (A) Fit of the full-length RfxMCR model in the representative cryo-EM densities. (B) Secondary structure topology of the full-length RfxMCR. (C and D) The density maps for the cofactor NADP+ and reaction intermediate MSA bound in the crystal structures of RfxMCR-N (C) and RfxMCR-C (D). The electron density maps are contoured as 2mFo-DFc at 2.0 σ level in gray. (E-F) The cryo-EM map (contoured at 1.5σ) of the substrate binding pocket of full-length RfxMCR incubated with NADP+ (E, 4.0 Å resolution) or malonyl-CoA (F, 4.2 Å resolution). The positions of NADP+ and malonyl-CoA are shown as green and magenta outlines to show the lack of cryo-EM densities. [file mbio.03233-22-s0003.tif]

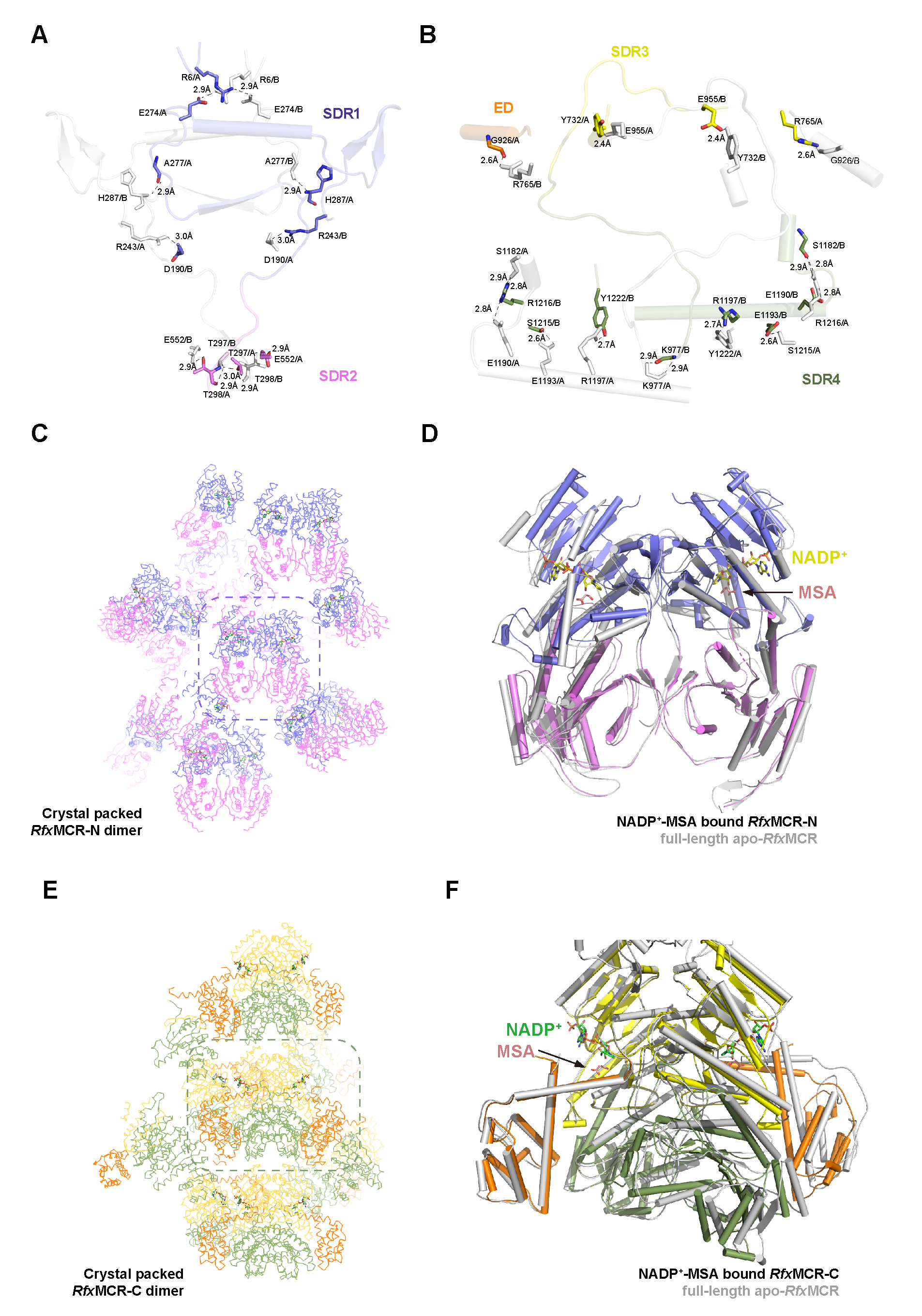

Supplement: Fig. S4 — Crystal packing and dimer interface of the NADP+–MSA-bound RfxMCR-N and RfxMCR-C, and the superimpositions with full-length RfxMCR. (A and B) The N- (A) and C-terminal (B) dimer interface of the full-length RfxMCR. The amino acid residues involved in forming hydrogen bonding interactions are shown in stick models, the hydrogen bonds are shown in dashed lines and labeled with distances. One subunit in the homodimer is colored in gray. (C and E) Crystal packing of the NADP+–MSA-bound RfxMCR-N (C) and RfxMCR-C (E). A homodimer (indicated with dashed line) was identified in the crystal packing. (D and F) Superimpositions of the NADP+–MSA-bound RfxMCR-N (D) and RfxMCR-C (F) with the full-length RfxMCR (gray). The SDR1, SDR2, SDR3, SDR4 and ED domains are colored in blue, violet, yellow, sage and orange, respectively. The bound NADP+ (yellow in SDR1 and green in SDR3) and MSA (salmon) are shown in stick models. [file mbio.03233-22-s0004.tif]

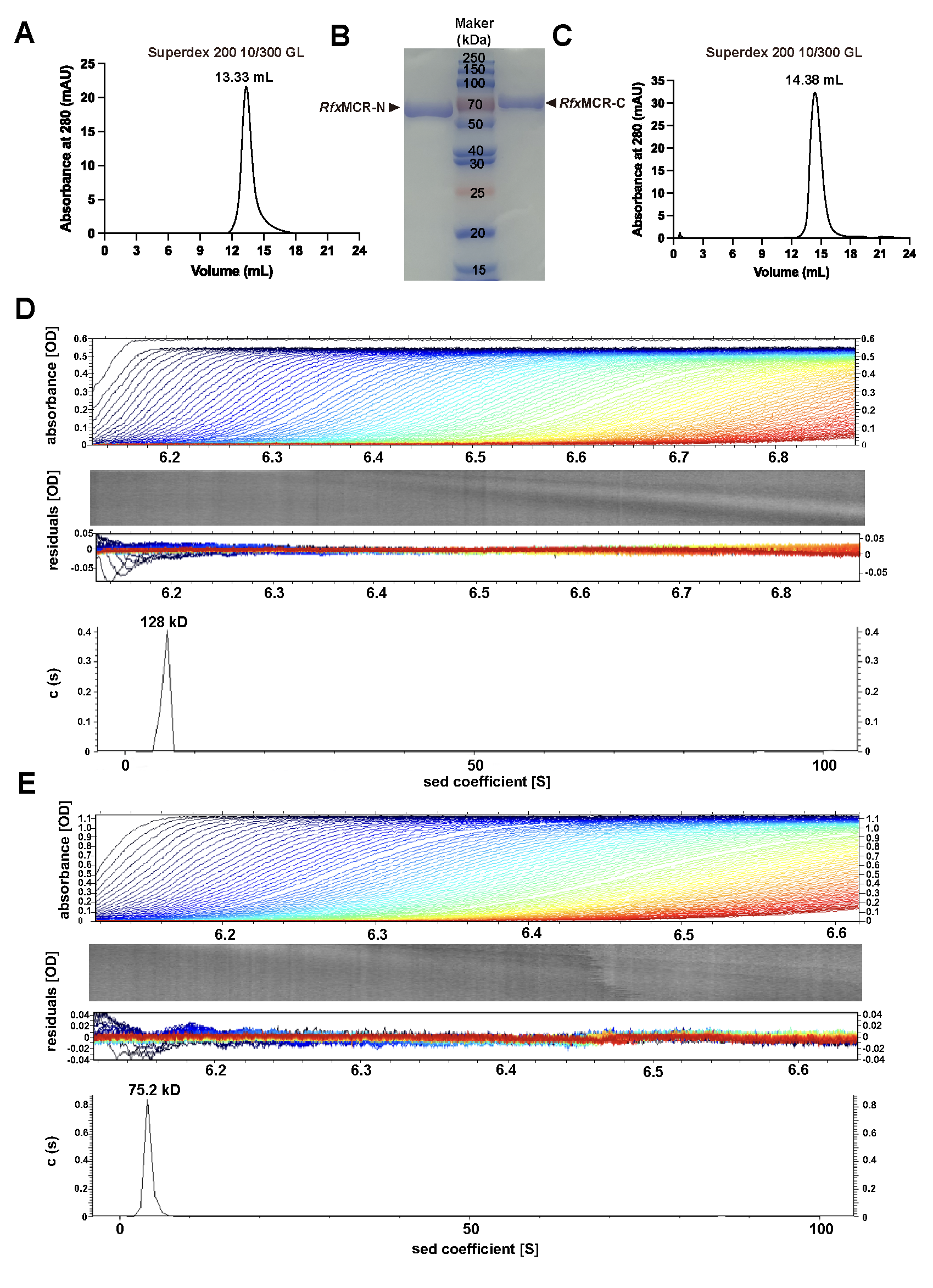

Supplement: Fig. S5 — Purification and analytic ultracentrifugation (AUC) of the RfxMCR-N and RfxMCR-C. (A-C) Gel filtration analyses (A and C) and SDS-PAGE (B) of the RfxMCR-N (A) and RfxMCR-C (C). The absorption of the RfxMCR-N (A) and RfxMCR-C (C) at 280 nm against the elution volume (mL) were recorded. (D-E) Sedimentation velocity analytical ultracentrifugation (AUC) of the RfxMCR-N (D) and RfxMCR-C (E). The upper panel shows the raw data from the time-course measurement of absorbance of the sample at 280 nm along the sample cell length. The middle panel shows residuals after fitting the data to the continuous size-distribution model. The lower panel shows the continuous mass distribution for the calculated solution. The curve indicates that the RfxMCR-N (D) exists as a homodimer in solution (the calculated MW of the monomer is 63.4 kDa), and the RfxMCR-C (E) exists as a monomer in solution (the calculated MW of the monomer is 72.9 kDa). [file mbio.03233-22-s0005.tif]

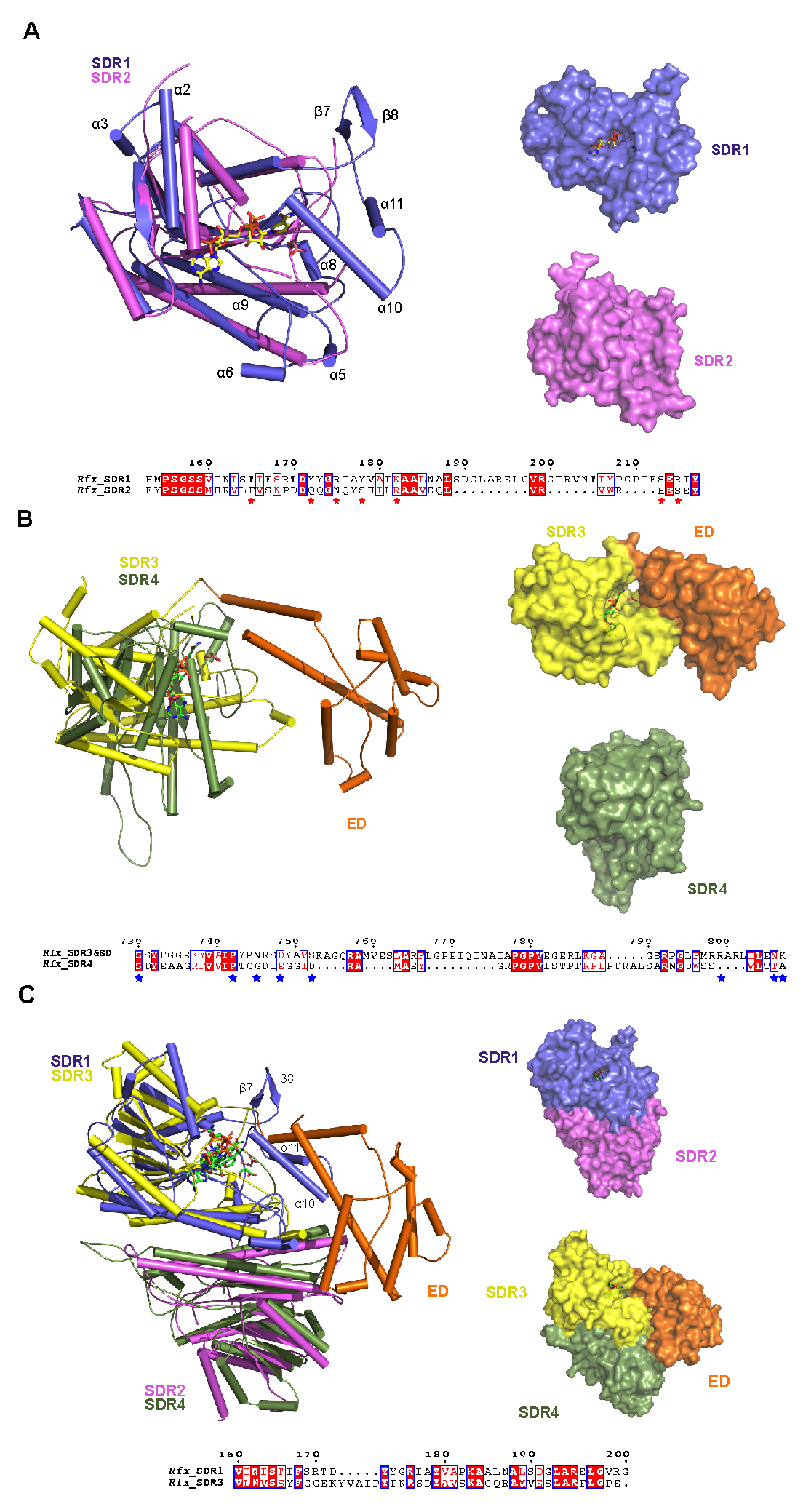

Supplement: Fig. S6 — Structural comparisons of the catalytic and non-catalytic SDR domains in RfxMCR. (A) Superimposition of the NADP+–MSA-bound SDR1 domain (blue) with the non-catalytic SDR2 domain (violet) gives a RMSD at 6.680 Å. The NADP+ (yellow) and MSA (salmon) are shown in stick models. The NADP+–MSA-bound SDR1 (right top) contains typical substrate binding pocket, whereas the non-catalytic SDR2 domain (right bottom) does not contain an optimal pocket for substrate binding. Sequence alignment of the SDR1 and SDR2 showed that the SDR2 does not contain conserved amino acid residues for substrate binding. The amino acid residues for substrate binding and catalysis are indicated with red stars. (B) Superimposition of the NADP+–MSA-bound SDR3 domain (yellow for SDR3 and orange for the ED) with the non-catalytic SDR4 domain (sage) gives a RMSD at 18.511 Å. The SDR3 (right top) but not SDR4 domain (right bottom) contains typical substrate binding pocket for NADP+–MSA binding. Sequence alignment of the SDR3 and SDR4 showed that the SDR4 does not contain conserved amino acid residues for substrate binding. The amino acid residues for substrate binding and catalysis in SDR3 are indicated by blue stars. (C) Superimposition of the NADP+–MSA-bound RfxMCR-N and RfxMCR-C structures. Surface presentation of RfxMCR-N (right top) and RfxMCR-C (right bottom) indicated distinct conformations of the substrate binding pockets. Sequence alignment of the RfxMCR-N and RfxMCR-C showed that the catalytic SDR1 and SDR3 domains contain strictly conserved amino acid residues for substrate binding and catalysis. [file mbio.03233-22-s0006.tif]

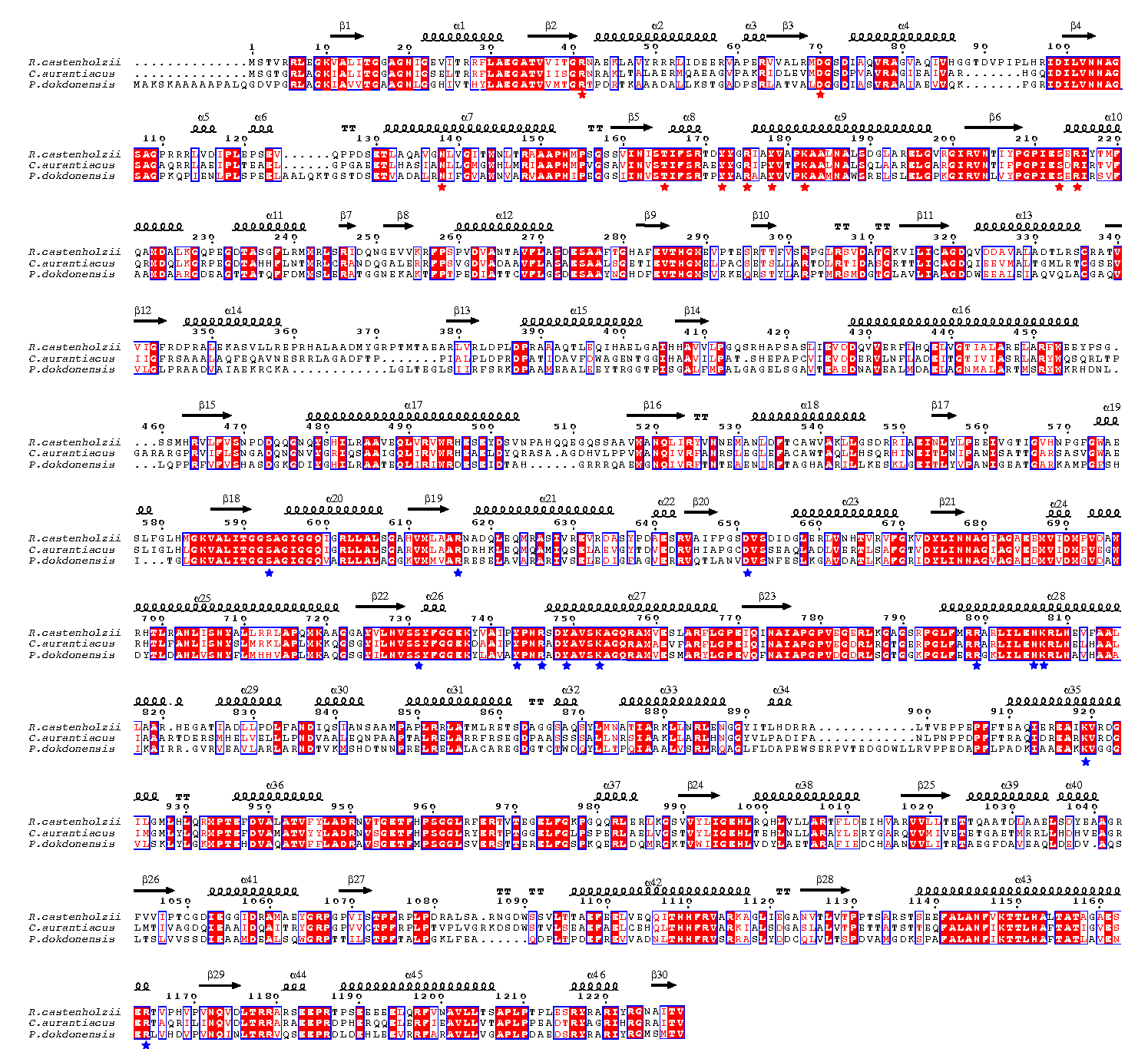

Supplement: Fig. S7 — Structure-based sequence alignment of the bi-functional MCRs from Roseiflexus castenholzii (RfxMCR, WP_012121415.1), Chloroflexus aurantiacus (CfxMCR, WP_012258473.1) and Porphyrobacter dokdonensis (PdMCR, WP_068862557.1). The secondary structures of RfxMCR are indicated on top of the sequence. The catalytic and substrate binding residues of RfxMCR-N and RfxMCR-C are indicated by red and blue stars, respectively. The YXXRXXY motifs are present in both the catalytic and substrate-binding sites of RfxMCR-N (Y172YGRIAY178) and RfxMCR-C (Y743PNRSDY749), in which the underlined letters represent the non-conserved amino acid residues. [file mbio.03233-22-s0007.tif]

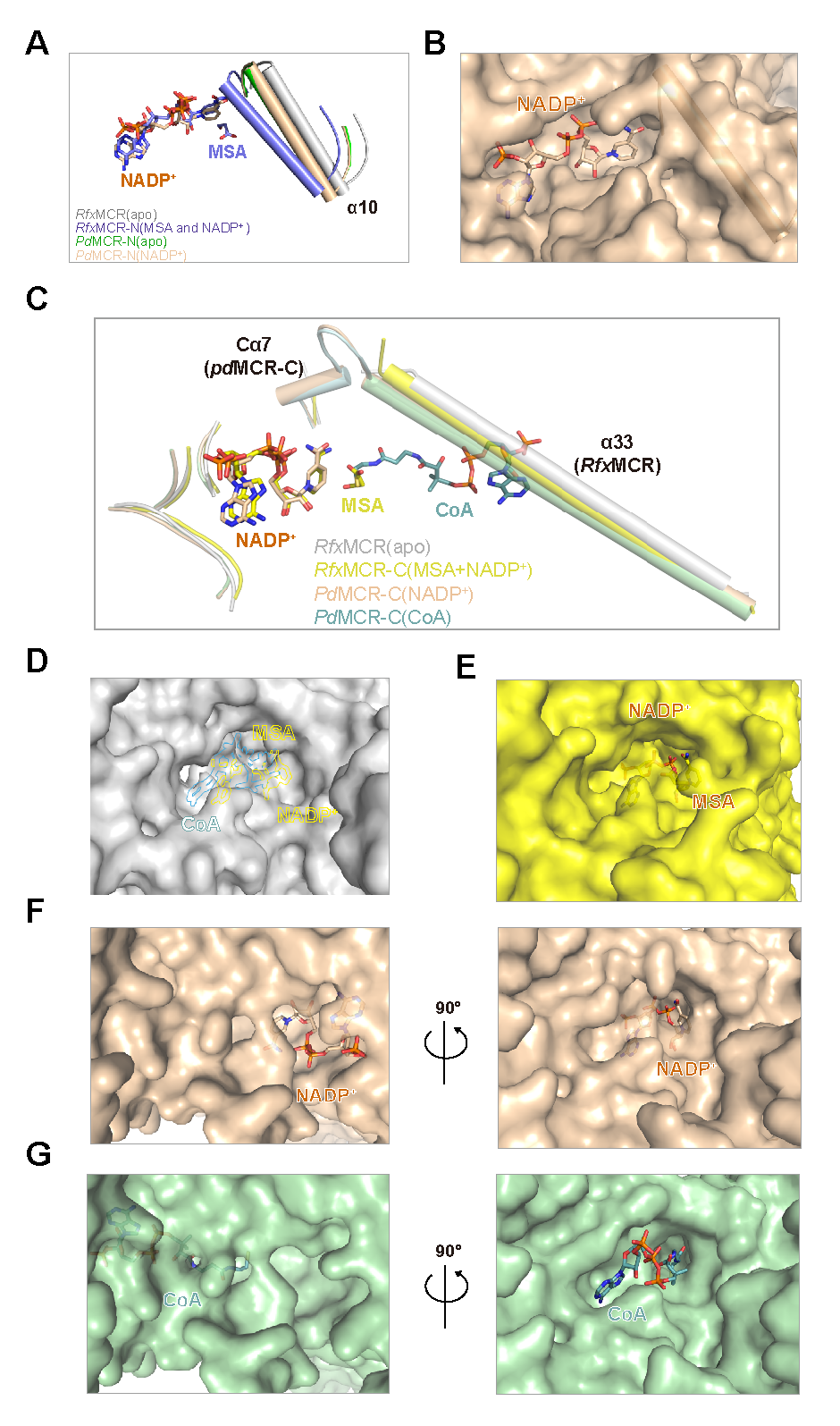

Supplement: Fig. S8 — Conformational changes of RfxMCR-N and RfxMCR-C in binding with NADP+–MSA. (A) Superimposition of the substrate binding pocket of NADP+–MSA-bound RfxMCR-N (blue) with that of full-length RfxMCR (gray), apo- (green) and NADP+-bound (wheat) PdMCR-N. (B) The substrate binding pocket of the NADP+-bound PdMCR-N is shown in surface. The NADP+ is shown in stick models. (C) Superimposition of the substrate binding pockets of NADP+–MSA-bound RfxMCR-C (yellow) with that of full-length RfxMCR (gray), the NADP+-bound (wheat) and CoA-bound (pale green) PdMCR-C. The α33 helix covering the CoA binding site is shown in ribbon, and the NADP+, MSA and CoA are shown in stick models. (D) Surface presentation of the substrate binding pocket of full-length RfxMCR (gray), the positions for binding the CoA (cyan), NADP+ and MSA (yellow) are shown and outlined. (E) Surface presentation of the substrate binding pocket of NADP+–MSA-bound RfxMCR-C (yellow). (F and G) Surface presentation of the substrate binding pocket of the NADP+-bound (F, wheat) and CoA-bound (G, pale green) PdMCR-C. [file mbio.03233-22-s0008.tif]

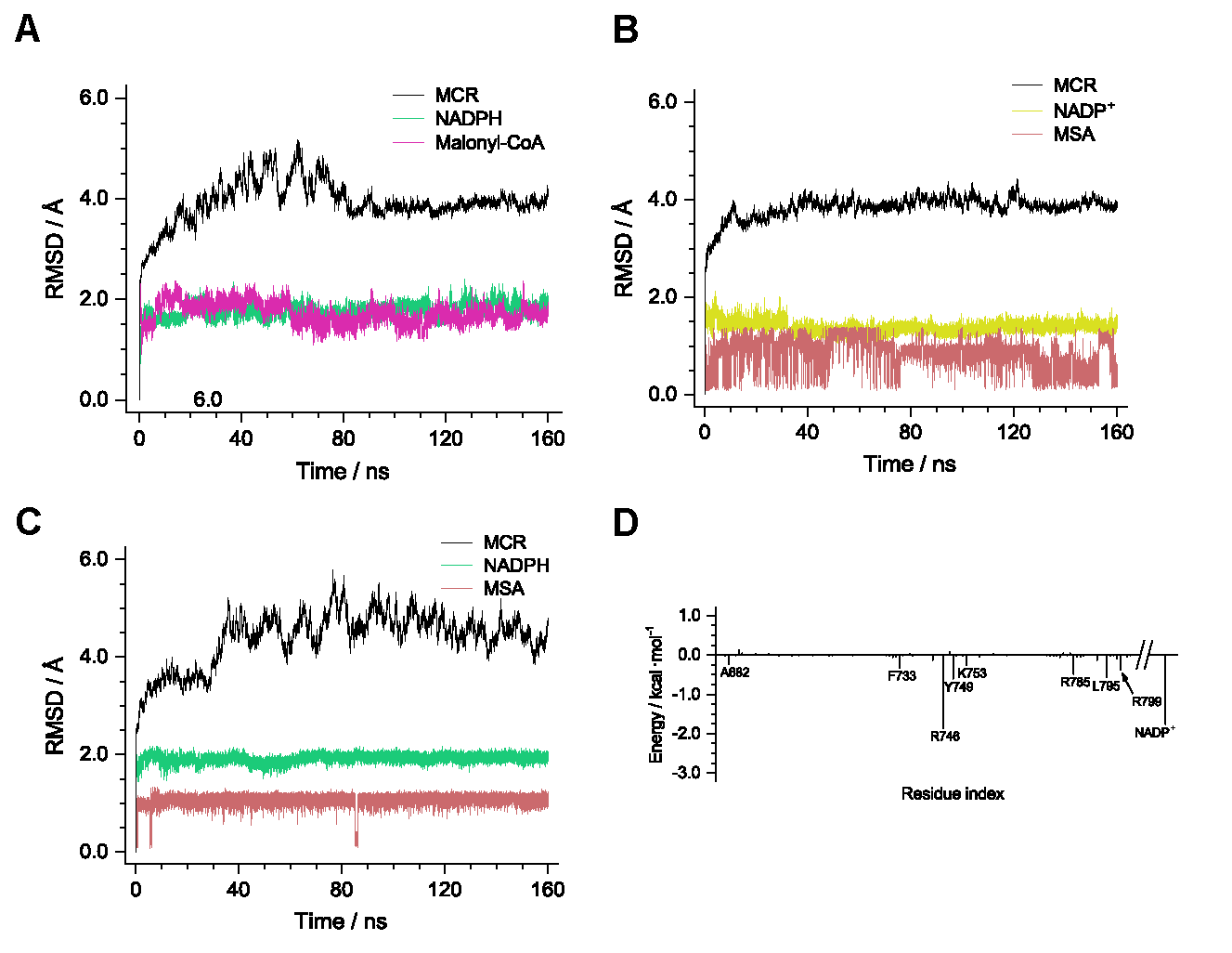

Supplement: Fig. S9 — Molecular dynamics (MD) simulations of the full-length RfxMCR. (A-C) MD simulations of the full-length RfxMCR in complex with malonyl-CoA and NADPH (A), NADP+-MSA (B) and NADPH-MSA (C). (A) Root-mean-square deviations (RMSDs) of full-length RfxMCR (black), the bound cofactor NADPH (green), and substrate malonyl-CoA (magenta) during MD simulations were plotted against the simulation time (ns). (B) RMSDs of full-length RfxMCR (black), the bound cofactor NADP+ (yellow), and intermediate MSA (salmon) during MD simulations were plotted against the simulation time (ns). (C) RMSDs of full-length RfxMCR (black), the bound cofactor NADPH (green), and intermediate MSA (salmon) during MD simulations were plotted against the simulation time (ns). (D) The per-residue decomposition of the binding free energies of full-length RfxMCR in binding with MSA and NADP+. [file mbio.03233-22-s0009.tif]

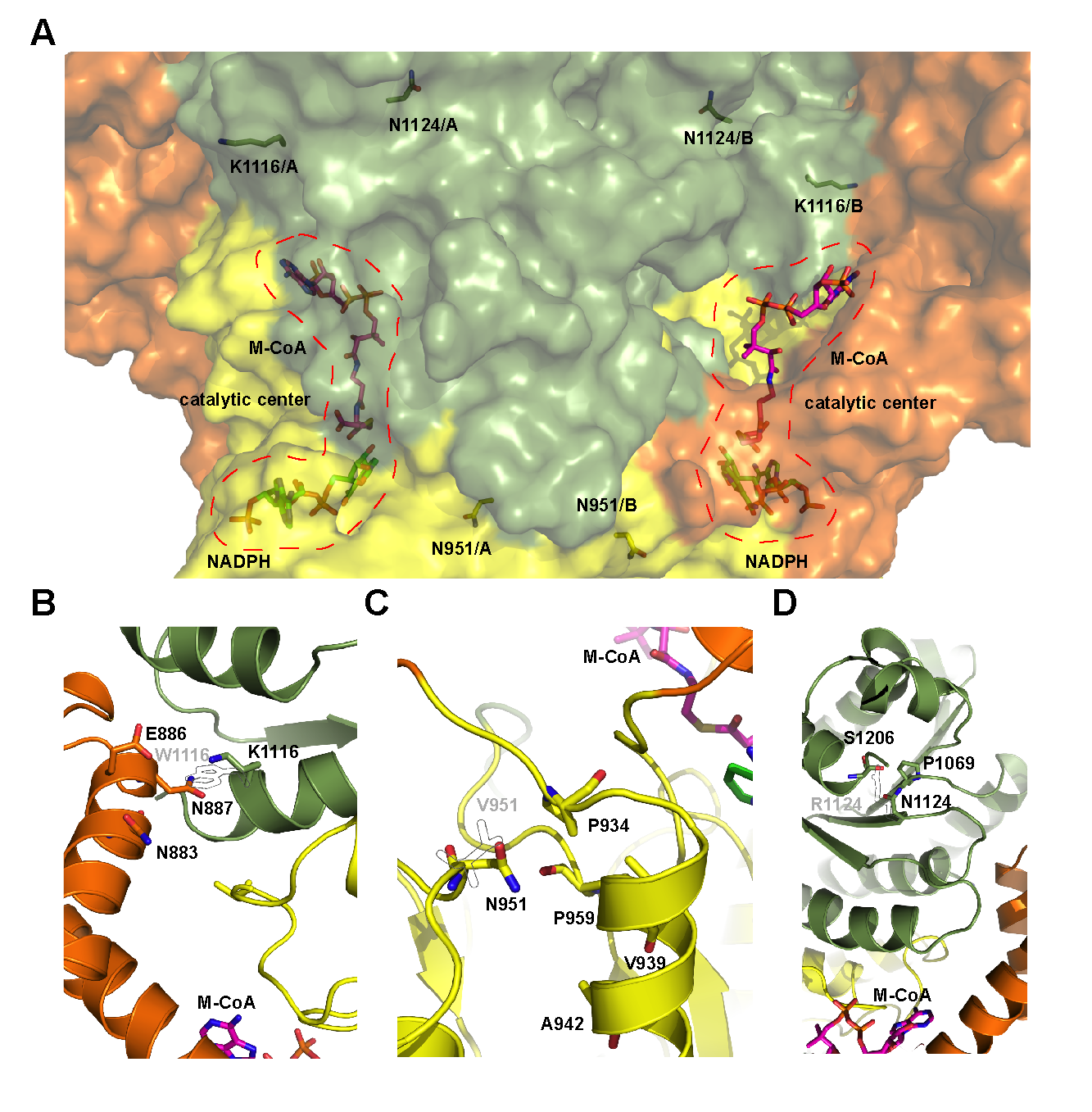

Supplement: Fig. S10 — Spatial organization of RfxMCR amino acid residues that are corresponding to the hot spot mutations of Chloroflexus aurantiacus MCR (CfxMCR). (A) Distribution of the amino acid residues that are corresponding to the hot spot mutations in CfxMCR. The catalytic centers of RfxMCR are indicated with bound substrate malonyl-CoA (magenta) and cofactor NADPH (green), the SDR3 (yellow), SDR4 (sage) and ED (orange) domains are shown in surface, and the amino acid residues are shown in stick models. (B-D) Spatial organizations of the amino acid residues Lys1116 (B), Asn951 (C) and Asn1124 (D) that are corresponding to the hot spot mutations K1106W, N940V and S1114R of CfxMCR. The amino acid residues in RfxMCR are shown in stick models, and the mutations in CfxMCR are shown as gray outline. [file mbio.03233-22-s0010.tif]
